# Visualization methods for differential expression analysis

**DOI:** 10.1186/s12859-019-2968-1

**Published:** 2019-09-06

**Authors:** Lindsay Rutter, Adrienne N. Moran Lauter, Michelle A. Graham, Dianne Cook

**Affiliations:** 1 Bioinformatics and Computational Biology Program, Iowa State University, Ames, USA; 2 USDA-ARS, Corn Insects and Crop Genetics Research Unit, Ames, USA; 3 Department of Agronomy, Iowa State University, Ames, USA; 4 Econometrics and Business Statistics, Monash University, Clayton VIC, Australia

**Keywords:** Interactive, RNA-sequencing, Statistical graphics, Visualization

## Abstract

**Background:**

Despite the availability of many ready-made testing software, reliable detection of differentially expressed genes in RNA-seq data is not a trivial task. Even though the data collection is considered high-throughput, data analysis has intricacies that require careful human attention. Researchers should use modern data analysis techniques that incorporate visual feedback to verify the appropriateness of their models. While some RNA-seq packages provide static visualization tools, their capabilities should be expanded and their meaningfulness should be explicitly demonstrated to users.

**Results:**

In this paper, we 1) introduce new interactive RNA-seq visualization tools, 2) compile a collection of examples that demonstrate to biologists *why* visualization should be an integral component of differential expression analysis. We use public RNA-seq datasets to show that our new visualization tools can detect normalization issues, differential expression designation problems, and common analysis errors. We also show that our new visualization tools can identify genes of interest in ways undetectable with models. Our R package “bigPint” includes the plotting tools introduced in this paper, many of which are unique additions to what is currently available. The “bigPint” website is located at https://lindsayrutter.github.io/bigPint and contains short vignette articles that introduce new users to our package, all written in reproducible code.

**Conclusions:**

We emphasize that interactive graphics should be an indispensable component of modern RNA-seq analysis, which is currently not the case. This paper and its corresponding software aim to persuade 1) users to slightly modify their differential expression analyses by incorporating statistical graphics into their usual analysis pipelines, 2) developers to create additional complex and interactive plotting methods for RNA-seq data, possibly using lessons learned from our open-source codes. We hope our work will serve a small part in upgrading the RNA-seq analysis world into one that more wholistically extracts biological information using both models and visuals.

**Electronic supplementary material:**

The online version of this article (10.1186/s12859-019-2968-1) contains supplementary material, which is available to authorized users.

## Background

RNA-sequencing (RNA-seq) uses next-generation sequencing (NGS) to estimate the quantity of RNA in biological samples at given timepoints. In recent years, decreasing cost and increasing throughput has rendered RNA-seq an attractive form of transcriptome profiling. Prior to RNA-seq, gene expression studies were performed with microarray techniques, which required prior knowledge of reference sequences. RNA-seq does not have this limitation, and has enabled a new range of applications such as *de novo* transcriptome assembly [1] and detection of alternative splicing processes [2, 3]. Coupled with its high resolution and sensitivity, RNA-seq is revolutionizing our understanding of the intricacies of eukaryotic transcriptomes [4, 5].

One common format of RNA-seq data is a matrix containing mapped read counts for *n* rows of genes and *p* columns of samples. These mapped read counts provide gene expression level estimations across samples. Researchers often conduct RNA-seq studies to identify differentially expressed genes (DEGs) between treatment groups. In most popular RNA-seq analysis packages, this objective is approached with models, such as the negative binomial model [6–9] and linear regression models [10].

Initially, it was widely claimed that RNA-seq produced unbiased data that did not require sophisticated normalization [4, 11, 12]. However, numerous studies have since revealed that RNA-seq data is replete with biases and that accurate detection of DEGs is not a negligible task. Problems that complicate RNA-seq data analysis include nucleotide and read-position biases [13], biases related to gene lengths and sequencing depths [14, 15], biases introduced during library preparation [16], and confounding combinations of technical and biological variability [17].

In light of these complications, researchers should analyze RNA-seq data like they would any other biased multivariate data. Solely applying models to such data is problematic because models hold assumptions that must be verified to ensure statistical soundness. Fortunately, data visualization enables researchers to see patterns and problems they may not otherwise detect with traditional modeling. As a result, the most effective approach to data analysis is to iterate between models and visuals, and enhance the appropriateness of applied models based on feedback from visuals [18]. With differential expression data, we primarily want to compare the variability between replicates and between treatment groups. This is visually best achieved by drawing the mapped read count distributions across all genes and samples. To our knowledge, the few plotting tools offered in popular RNA-seq packages do *not* often allow users to effectively view their data in this manner.

In this paper, we strive to remedy this problem by highlighting the utility of new and effective differential expression plotting tools. We use real RNA-seq data to show that our tools can detect normalization problems, DEG designation problems, and common errors in the analysis pipeline. We also show that our tools can identify genes of interest that cannot otherwise be obtained by models. We emphasize that interactive graphics should be an indispensable component of modern RNA-seq analysis. Here, we do not propose that users drastically change their approach to differential expression analysis. Instead, we propose that users simply modify their approach to differential expression analysis by assessing the sensibility of their models with multivariate graphical tools, namely with parallel coordinate plots, scatterplot matrices, and litre plots.

## Results

### Parallel coordinate plots

Parallel coordinate plots are essential to inform the relationships between variables in multivariate data. A parallel coordinate plot draws each row (gene) as a line. For a given gene, two samples with similar read counts will have a flat connection and two samples with dissimilar read counts will have a sloped connection. The ideal dataset has more variability between treatments than between replicates. Researchers can quickly confirm this with a parallel coordinate plot: There should be flat connections between replicates but crossed connections between treatments.

There are several packages within the Bioconductor software [19] that provide graphics for RNA-seq data analysis [20]. Two of the most common graphic techniques are side-by-side boxplots and Multidimensional Scaling (MDS) plots [9, 21–23]. Unfortunately, these plots can hide problems that still exist in the data even after normalization and that could be better detected with parallel coordinate plots.

**Fig. 1:**
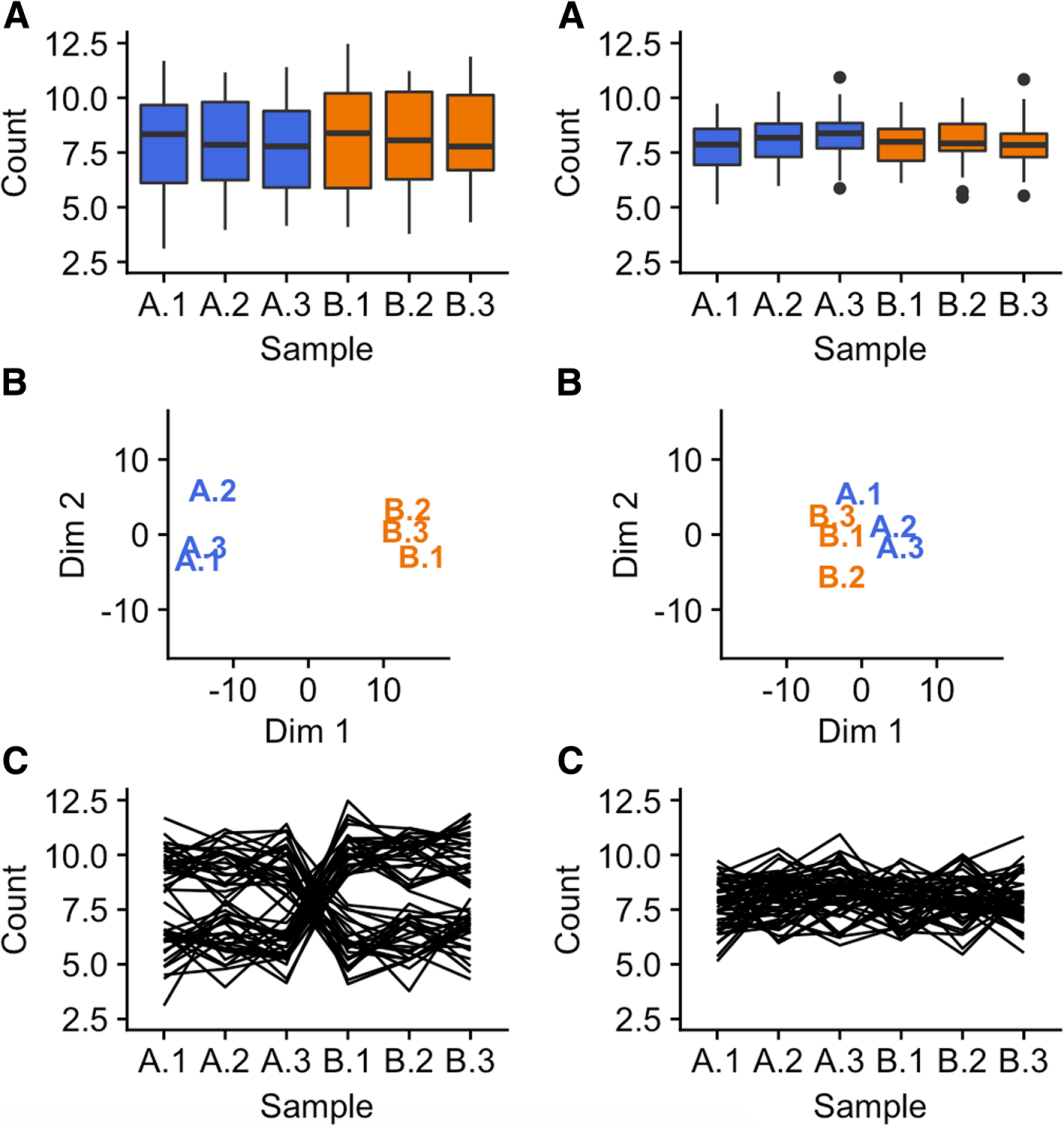
Comparison of plotting methods using simulated data. One simulated dataset is shown on the left half and another simulated dataset is shown on the right half of the figure. The parallel coordinate plots (subplots C) show a critical difference at the gene-level between the datasets. Namely, the left dataset is composed of genes with small replicate variation and large treatment group variation (suggesting DEGs), while the right dataset is composed of genes with similar variation between replicates and treatment groups (not suggesting DEGs). We cannot see this gene-level difference with the boxplots and MDS plots

Figure 1 exemplifies this problem for two *simulated* datasets, one displayed on the left half and the other displayed on the right half of the figure. Each dataset contains two treatment groups (A and B) with three replicates. The side-by-side boxplots (subplots A) both show fairly consistent medians across the six samples in the left and right dataset; the most prominent difference is the smaller interquartile ranges in the right dataset. The left MDS plot separates the treatment groups distinctively; the right MDS plot suggests a similar separation but in a much subtler manner (subplots B). In addition, the first replicate from treatment A appears as an outlier in the right MDS plot.

While the boxplots and MDS plots provide useful information, the parallel coordinate plots (subplots C) show an additional meaningful difference between the left and right datasets. The left dataset has consistent (level) lines between replicates and inconsistent (crossed) lines between treatment groups. This suggests that some of the genes (lines) have consistently low values for treatment group A and consistently high values for treatment group B, while some genes have the opposite phenomenon. As a result, the majority of the plotted genes may be DEG candidates. In contrast, the right dataset does not possess this ideal structure and suggests that the majority of its genes may not be DEG candidates. We could not see this important distinction as clearly using the side-by-side boxplots or the MDS plots because they only provide data summarization at the sample resolution, while the parallel coordinate plots show the sample connections for each gene in the data.

**Fig. 2:**
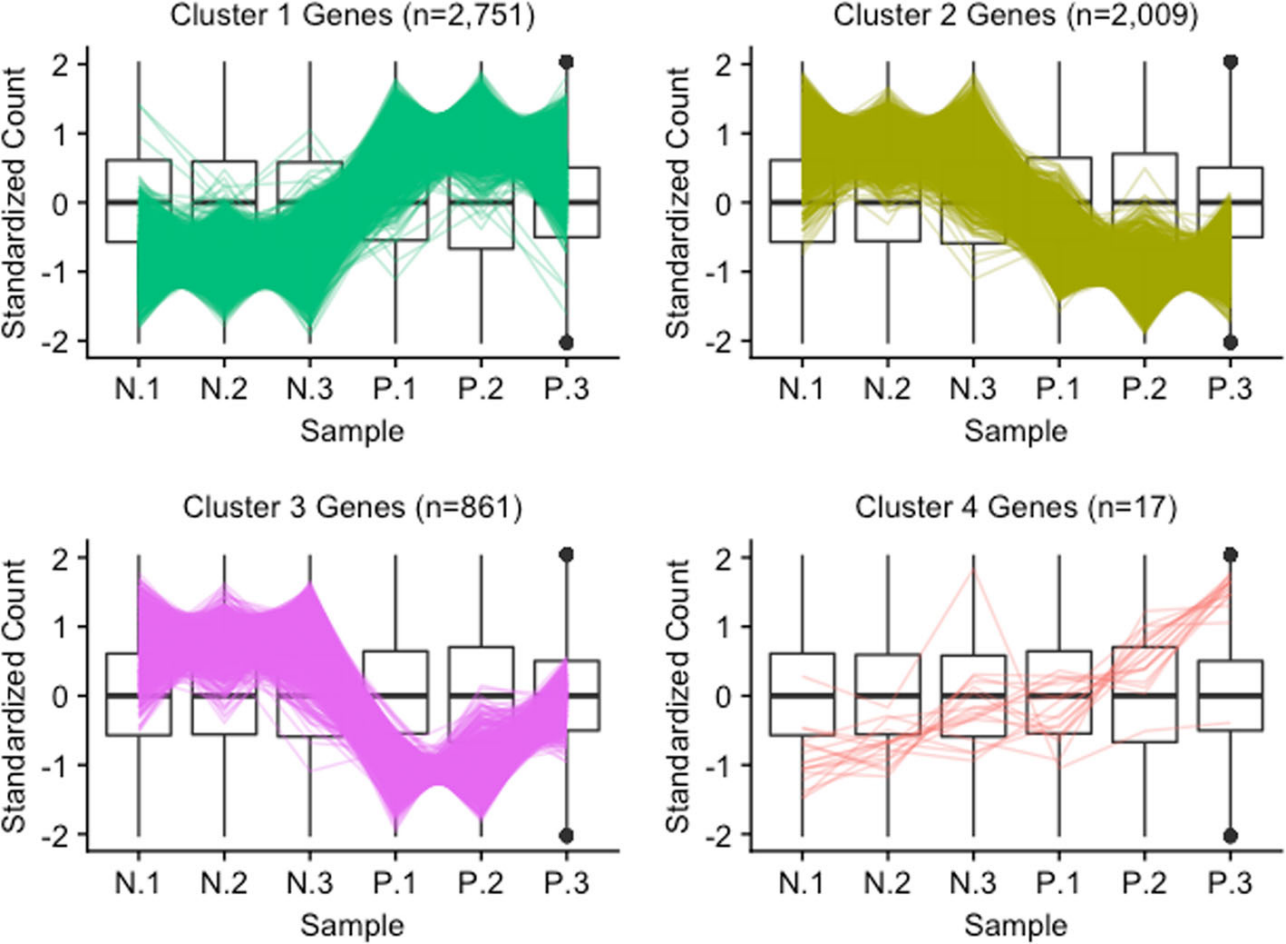
Parallel coordinate plots of clustered significant genes in the soybean iron metabolism data. Parallel coordinate plots of significant genes after hierarchical clustering of the soybean iron metabolism data [24]. We can quickly confirm that Clusters 1 and 2 show the typical pattern for significant genes. Cluster 4 does not distinctively show the usual profile for significant genes. Cluster 3 looks similar to Cluster 2, except for unexpectedly large P.3 values

Please note that the example above was simulated for didactic purposes. We will now examine the application of parallel coordinate plots to real data from an RNA-seq study that compared soybean leaves after 120 min of iron-sufficient (group P) and iron-deficient (group N) hydroponic treatments [24]. We filtered genes with low means and/or variance, performed a hierarchical clustering analysis with a cluster size of four, retained only significant genes, and visualized the results using parallel coordinate lines (Fig. 2). For these visualizations, we standardized each gene to have a mean of zero and standard deviation of unity [25, 26]. Then, we performed hierarchical clustering on the standardized DEGs using Ward’s linkage. This process can divide large DEG lists into smaller clusters of similar patterns, which allows us to more effectively detect the various types of patterns within large DEG lists. We note that the number and quality of clusters can vary depending on the data.

The majority of significant genes were in Clusters 1 and 2, which for the most part captured the expected patterns of differential expression (consistent replicates and inconsistent treatments) in reverse directions. Only 17 significant genes belonged to Cluster 4 and they mostly showed messy patterns with low signal to noise ratios. Interestingly, Cluster 3 had a fairly large number of significant genes (*n*=861). These genes mostly showed clean differential expression profiles similar to Cluster 2 (large values for group N and small values for group P), except for unexpectedly large values for the third replicate of group P. The reasons for a different response by these genes on this replicate is unclear, but warrants further study.

### Scatterplot matrices

### Overview of scatterplot matrices

A scatterplot matrix is another effective multivariate visualization tool that plots read count distributions across all genes and samples. Specifically, it represents each row (gene) as a point in each scatterplot. With this method, users can quickly discover unexpected patterns, recognize geometric shapes, and assess the structure and association between multiple variables in a manner that is different from most common practices.

**Fig. 3:**
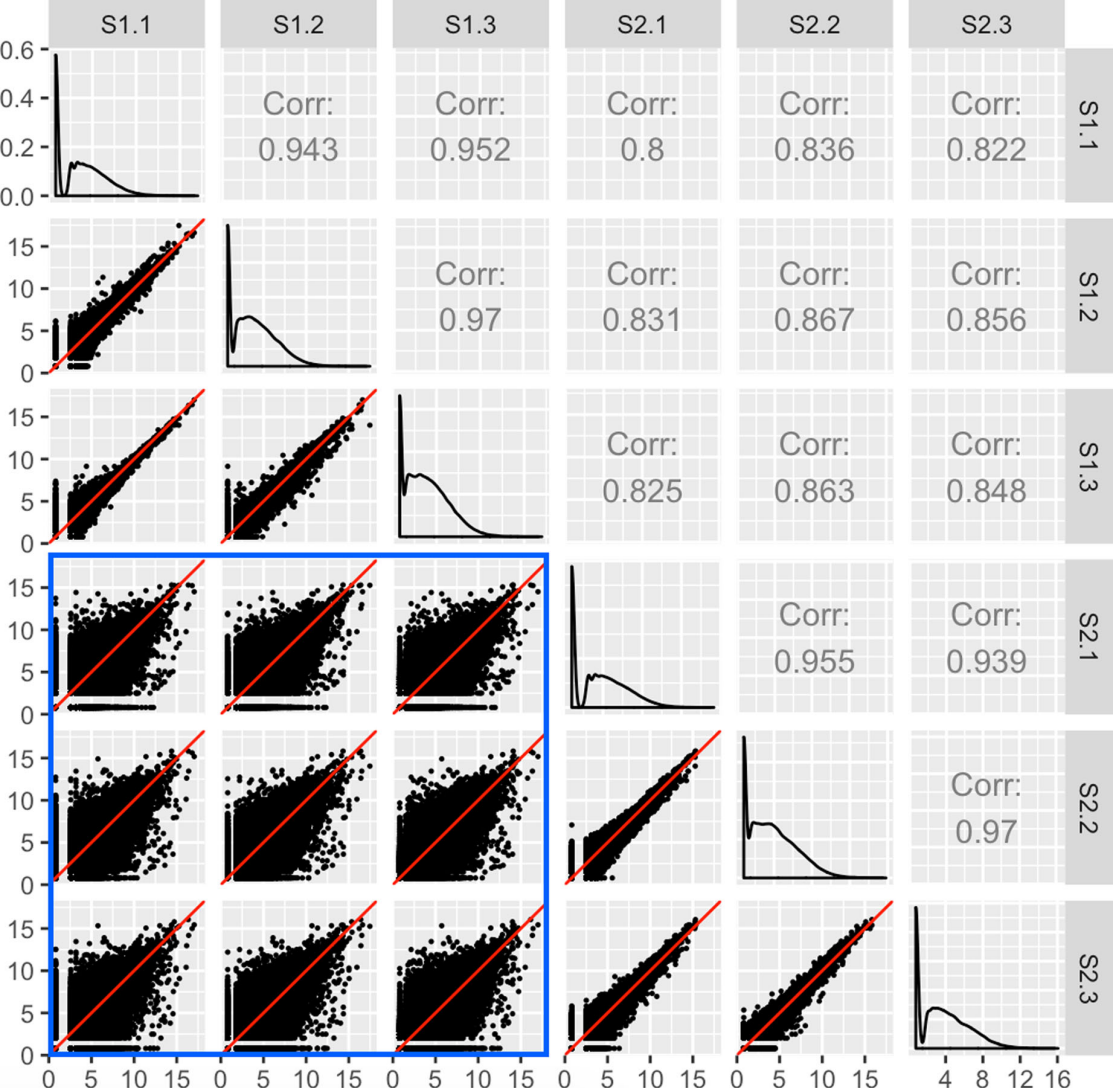
Expected structure of RNA-seq data plotted as a scatterplot matrix. Example of the expected structure of an RNA-seq dataset, using soybean cotyledon data from [27]. Within a given scatterplot, most genes (points) should fall along the *x=y* line. We should see genes deviate more strongly from the *x=y* line in treatment scatterplots (the nine scatterplots enclosed in the blue square) than in replicate scatterplots (the remaining six scatterplots)

Clean data would be expected to have larger variability between treatment groups than between replicates. As Fig. 3 shows, researchers can quickly confirm this with a scatterplot matrix. Within each scatterplot, most genes should fall along the *x=y* line (in red) as we expect only a small proportion of them to show differential expression between samples. However, a fraction of the genes should have lower variability between replicates than between treatments, and so we should expect the spread of the scatterplot points to fall more closely along the *x=y* relationship between replicates than between treatments. Indeed, in Fig. 3, we created a scatterplot matrix for a public RNA-seq dataset that contains three replicates for two developmental stages of soybean cotyledon (S1 and S2) [27]. We can immediately verify that the nine scatterplots between treatment pairs (the bottom-left corner of the matrix encased in the blue square) have more spread around the *x=y* line than the six scatterplots between replicate pairs.

After confirming this expected trend, users can use the scatterplot matrix to focus on subsets of genes: Outlier genes that deviate from the *x=y* line in replicate scatterplots might be problematic, whereas outlier genes that deviate from the *x=y* line in treatment scatterplots might be DEGs. In order to achieve this functionality, the plots must be rendered interactive. This way, users can hover over and click on gene subsets of interest and view their patterns from multiple perspectives while also obtaining their identifiers.

Notice that each gene in our data is plotted once in each of the 15 scatterplots. With 73,320 genes in our data, more than one million points must be plotted. Rendering all points interactive would slow down the interactive capabilities of the plot. To solve this, we can tailor the geometric object of the scatterplots to be hexagon bins rather than points. This dramatically reduces the number of geometric objects to be plotted, and increases the interactivity speed.

The interactive version of Fig. 3 is available online [28]. Readers can read the “About" Tab to fully understand how to use the application. Essentially, the user can hover over a hexagon bin to see how many genes it contains. When the user clicks on a hexagon bin, the names of the genes are listed and superimposed as orange points across all scatterplots. The genes are also linked to a second plot that superimposes them as parallel coordinate lines on a side-by-side boxplot of *all* gene counts in the dataset. This interactivity and linking allows users to quickly examine genes of interest from multiple viewpoints superimposed onto the summary of *all* genes in the dataset.

### Assessing normalization with scatterplot matrices

There is still substantial discussion about the normalization of RNA-seq data, and the scatterplot matrix can be used to understand and assess various algorithms. To exemplify this point, we will use a publicly-available RNA-seq dataset on Saccharomyces cerevisiae (yeast) grown in YP-Glucose (YPD) [22]. The data contained four cultures from independent libraries that were sequenced using two library preparation protocols and either one or two lanes in a total of three flow-cells. This experimental design allowed researchers to examine various levels and combinations of technical effects (library preparation and protocol and flow cell) and biological effects (culture).

The four cultures (Y1, Y2, Y4, and Y7) were treated as biological replicates for which differential expression was not expected. Hence, the authors could establish a false positive rate in relation to the number of DEGs called between these groups. They then demonstrated that within-lane regression alone was insufficient in effectively removing biases. Instead, aggressive corrections for both within-lane (GC-content and gene length) and between-lane (count distribution and sequencing depth) biases were needed to effectively reduce the false-positive rate of DEG calls.

**Fig. 4:**
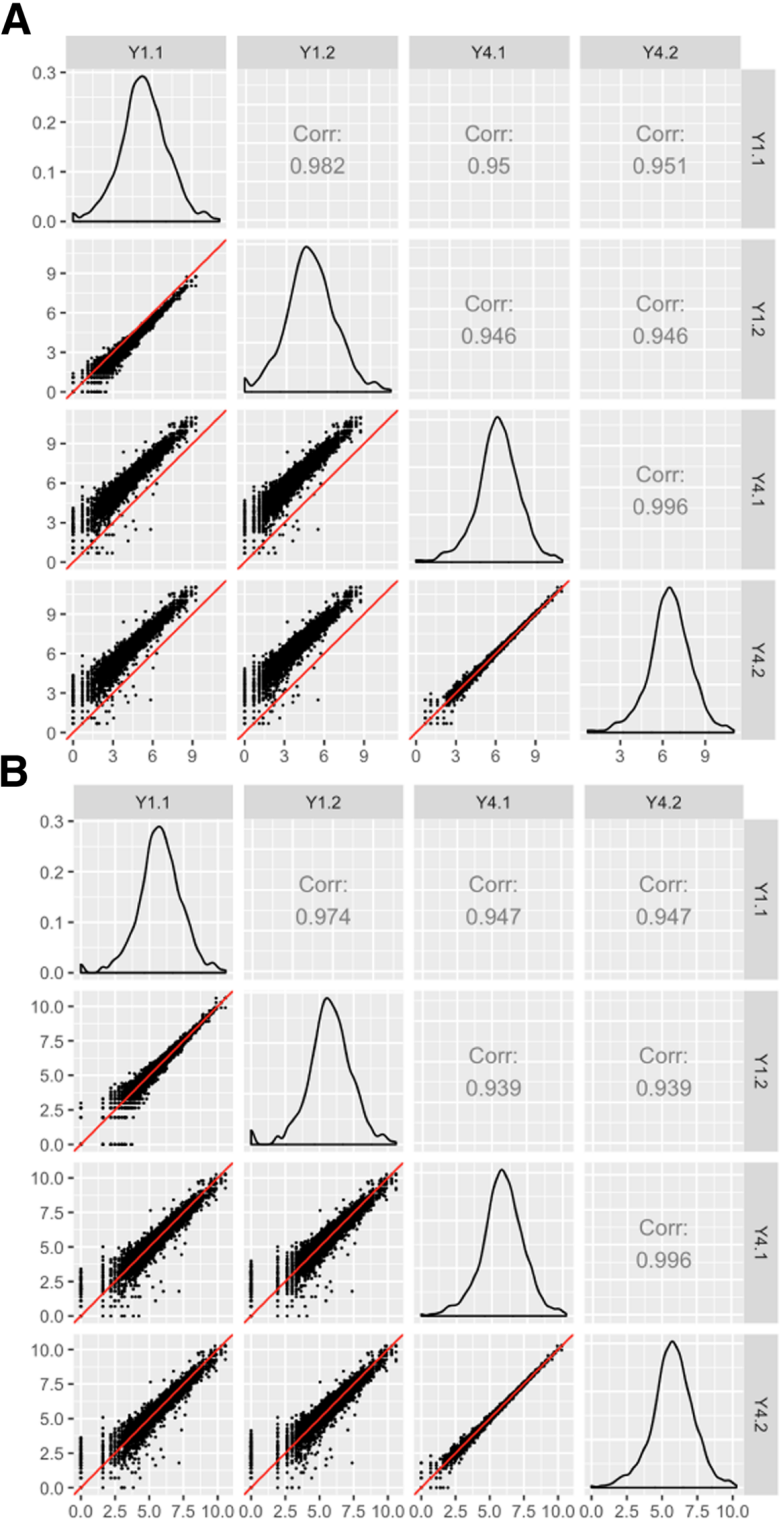
Assessing normalization of RNA-seq data using scatterplot matrices. Illustrating normalization checks with data from [22]. The collective deviation of genes from the *x=y* line instantly reveals that the RNA-seq dataset was not thoroughly normalized using within-lane normalization (subplot **a**). However, within-lane normalization followed by between-lane normalization sufficiently normalized the data (subplot **b**). The authors who developed these normalization methods showed that the later approach generated a lower false-positive DEG call rate in this dataset

Figure 4a shows the scatterplot matrix of the read counts from the Y1 and Y4 treatments after within-lane normalization. As we stated earlier, we expect most genes to show similar expression between samples, except for the handful that are differentially expressed. However, it is immediately clear that the data still was not sufficiently normalized as the distribution of genes is not centered around the *x=y* lines. In contrast, Fig. 4b shows the scatterplot matrix of the read counts from the Y1 and Y4 treatments after *both* within-lane and between-lane normalization, as was recommended by the authors due to its reduced false-positive rate. Indeed, the scatterplot matrix now follows the expected structure with most genes falling along the *x=y* line with thicker deviations from it between treatment groups than between replicate groups.

Additionally, we can also confirm from Fig. 4b that the read counts fall closer to the *x=y* line between the Y4 replicates (bottom-right scatterplot) than between the Y1 replicates (top-left scatterplot). This is expected because the Y1 replicates had additional technical variability as they used two different flow cells, whereas the Y4 replicates were from the same flow cell. As such, the scatterplot matrix can also be used to quickly inspect patterns of biological and technical variability in the dataset.

### Checking for common errors with scatterplot matrices

Irreproducibility is prevalent in high-throughput biological studies. A study in Nature Genetics surveyed eighteen published microarray expression analyses and reported that only two were exactly reproducible [29]. The extent of the problem has spawned a field called “forensic bioinformatics" whereby researchers attempt to reverse-engineer reported results back into the raw datasets simply to derive the methodologies used in published studies [30].

Even though irreproducibility is merely cumbersome when it masks methods, it is unquestionably hazardous when it masks errors. With regards to personalized medicine, for example, obscured errors may cause well-intentioned researchers to present evidence for drugs that are ineffective or even harmful to patients [30]. Forensic bioinformaticians who have actively investigated common errors in high-throughput biological studies have concluded that the largeness of the data itself may hinder our ability to detect errors [30]. They also discovered that the most common errors are simple errors, such as mixing up sample labels [30]. Collectively, these findings suggest that simple errors can be difficult to detect using common practices in high-throughput studies.

**Fig. 5:**
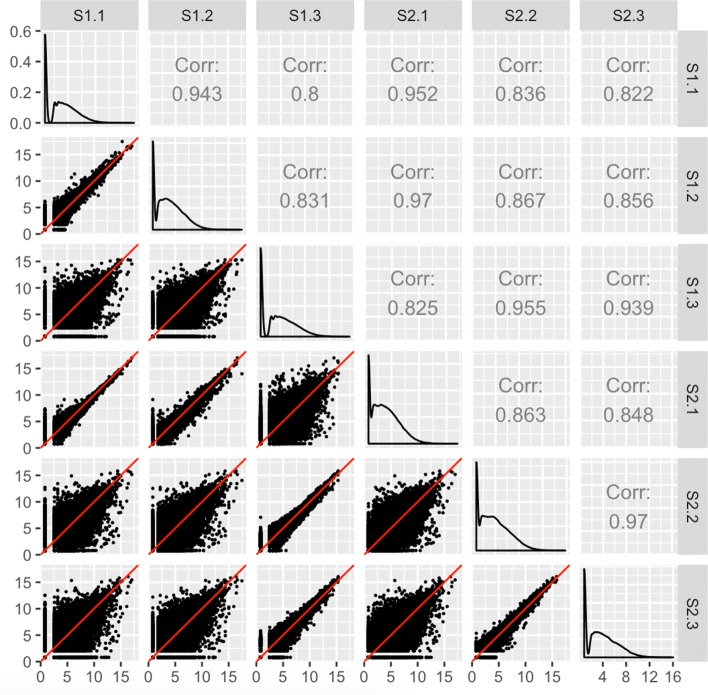
Checking common errors of RNA-seq data analysis using scatterplot matrices. As expected, the scatterplot matrix of the coytledon data [27] contains nine scatterplots with thicker distributions (should be treatment pairs) and six scatterplots with thinner distributions (should be replicate pairs). However, a subset of scatterplots unexpectedly show thicker distributions between replicate pairs and thinner distributions between treatment pairs. If we switch the labels of two suspicious samples (S1.3 and S2.1), the scatterplot matrix displays the anticipated structure we saw in Fig. 3. At this point, we have evidence that these two samples may have been mislabeled, and we may wish to confirm this suspicion and correct it before continuing with the analysis

**Fig. 6:**
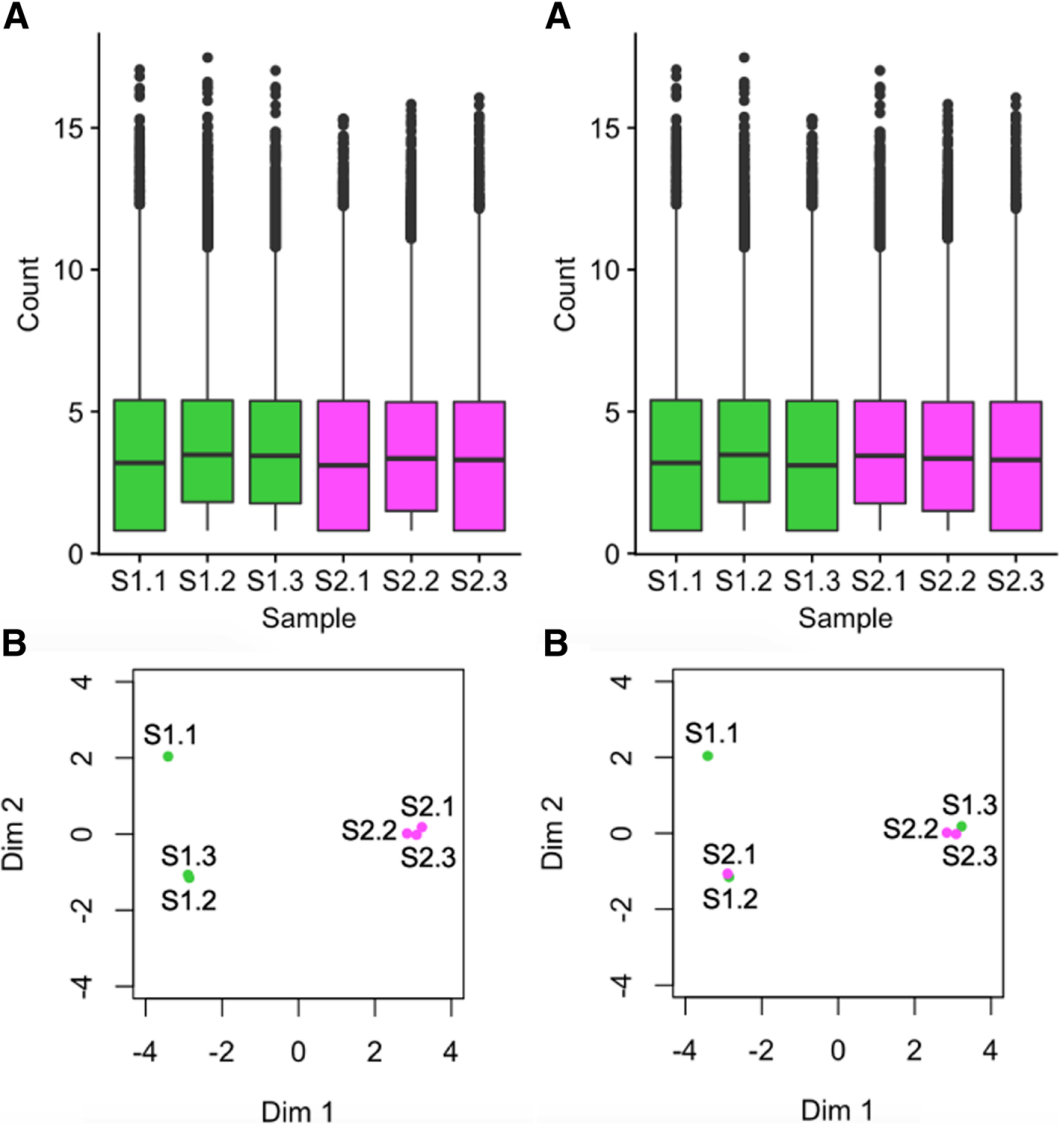
Checking common errors of RNA-seq data analysis using side-by-side boxplots and MDS plots. Side-by-side boxplots and MDS plots are popular plotting tools for RNA-seq analysis. This figure shows these traditional visualization methods applied to the soybean cotyledon data before sample switching (left half) and after sample switching (right half) [27]. We cannot suspect from the right boxplot that samples S1.3 and S2.1 have been swapped (subplots A). This is because all six samples have similar five number summaries. For the MDS plots, we do see a cleaner separation of the two treatment groups across the first dimension in the left plot than in the right plot (subplots B). However, taking into account the second dimension, both MDS plots contain three clusters, with sample S1.1 appearing in its own cluster. Without seeing one distinct cluster for each of the two treatment groups, it is difficult to suspect that samples S1.3 and S2.1 have been swapped in the right MDS plot (subplots B). We can only derive clear suspicion that the samples may have been switched by using less-popular plots that provide gene-level resolution like with the scatterplot matrix from Fig. 5

Fortunately, scatterplot matrices are a convenient tool to check for common errors like sample mislabeling. Figure 5 shows the resulting scatterplot matrix after we deliberately swapped the labels of the third replicate of the first treatment group (S1.3) with the first replicate of the second treatment group (S2.1) in the previously-mentioned cotyledon dataset [27]. We can immediately see that, as expected, there are nine scatterplots with thicker distributions around the *x=y* line and six scatterplots with thinner distributions around the *x=y* line. However, we notice that a subset of these thick and thin scatterplots appear outside of their expected locations given the expected variability between treatments versus replicates. Rearranging the columns of the two samples that appear suspicious in Fig. 5 would indeed lead us back to the clean-looking scatterplot matrix we saw in Fig. 3. The scatterplot matrix provides us convincing evidence of a mislabeling problem even down to the gene level, which cannot be confirmed with such detail using traditional plots like the boxplots and MDS plots before sample switching (left side of Fig. 6) and after sample switching (right side of Fig. 6). While this method can inform suspicious patterns in more detail than other means, users must still perform extra steps to determine if these patterns more likely relate to mislabeling or some real biological phenomenon. In the case of suspected mislabeling, the user would still need to substantiate this suspicion with decisive evidence and should only use the visualization as a guide.

### Finding unexpected patterns in scatterplot matrices

Most popular RNA-seq plotting tools display summaries about the read counts, such as fold change summaries, principal component summaries, five number summaries, and dispersion summaries. In contrast to this trend, scatterplot matrices display the non-summarized read counts for all genes. This trait allows for geometric shapes and patterns relevant to the read count distribution to be readily visible in the scatterplot matrix.

**Fig. 7:**
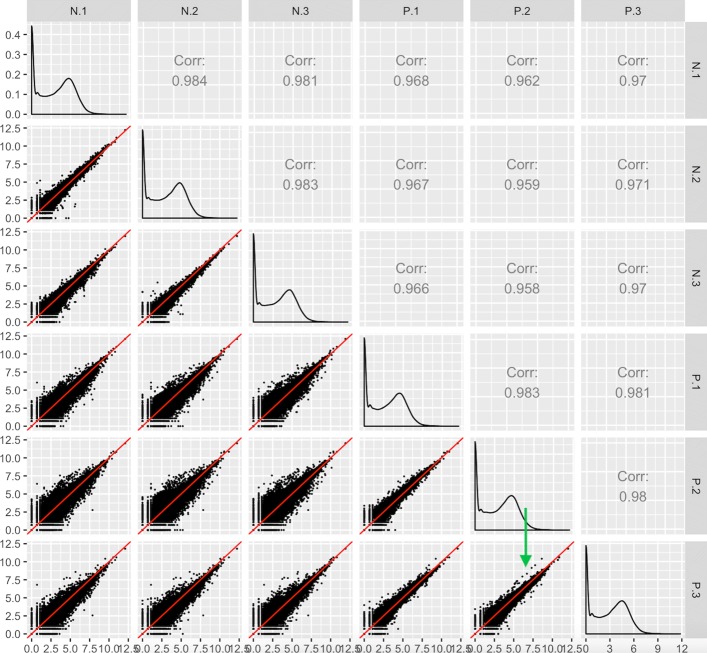
Finding unexpected patterns in RNA-seq data using scatterplot matrices. Scatterplot matrix of RNA-seq read counts from soybean leaves after exposure to iron-sufficient (treatment group P) and iron-deficient (treatment group N) hydroponic conditions [24]. We observe the expected structure of treatment pairs showing larger variability around the *x=y* line than replicate pairs. However, we notice a pronounced streak structure in the bottom-right scatterplot (green arrow) that compares two replicate samples from the iron-sufficient group. The genes in the streak structure have large read counts that deviate in a parallel fashion from the *x=y* line

An example of how geometric shapes in the scatterplot matrix can provide applicable information to researchers is shown in Fig. 7, which uses the iron-metabolism soybean dataset [24]. After normalizing the data, we see the expected pattern of a scatterplot matrix, with more variation around the *x=y* line between treatments than between replicates (Fig. 7). However, one streak structure in the bottom right scatterplot stands out. A small subset of transcripts between replicates of the iron-sufficient group sharply deviates from the *x=y* line. By interacting with the plot, we identified the five transcripts that deviated the most from the expected pattern, and searched for their putative functions. We discovered that these transcripts are reportedly involved in biotic and abiotic stress responses as well as the production of superoxides to combat microbial infections. It should be noted that these five transcripts did not reach significance unless the third replicate of the P group was removed. Therefore, these genes will still be reported as non-significant in this study.

**Fig. 8:**
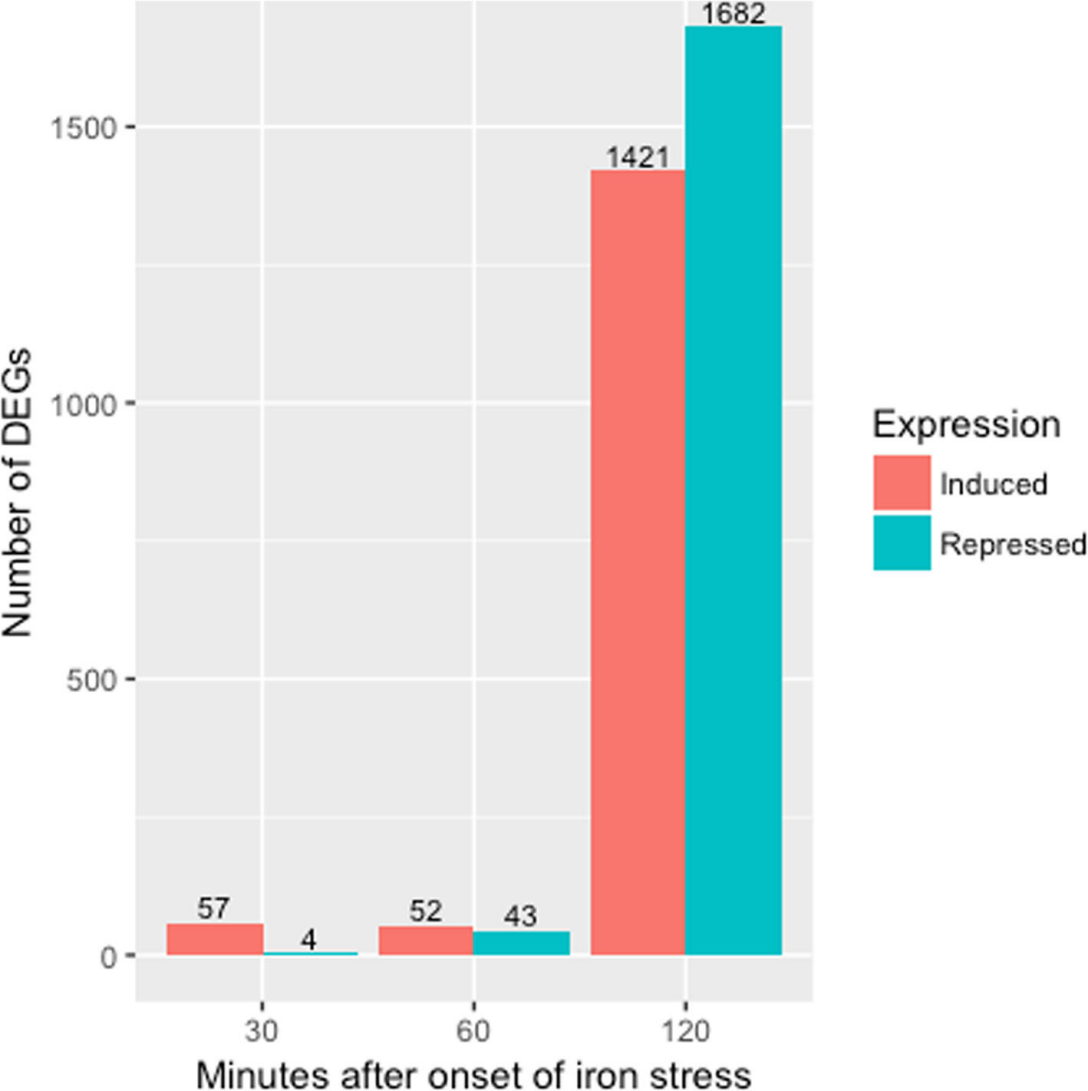
Gene expression responses across time points. The authors of the soybean iron metabolism study [24] determined the DEGs across three times points (30 min, 60 min, and 120 min) in the leaves after onset of iron sufficient and deficient hydroponic conditions. They used the same researcher to collect the samples in succession. One major finding from their study was a vaste change in gene expression responses between these three time points. As a result, the streak observed in the scatterplot matrix containing the subset of data at the 120 min time point (Fig. 7) may be due to the timing differences between replicate handling

Discussion with the authors of the study revealed that a lab biologist documented a clean data collection process. In the study, the authors determined the DEGs across three times points (30 min, 60 min, and 120 min) after exposure to the two iron condition levels. In order to reduce variability caused by plant handling by different researchers, the same researcher collected the samples in succession. One major finding from their study was a vast change in gene expression responses between these three time points (Fig. 8). In light of these discoveries, the authors tentatively suggest that the streak of genes shown in Fig. 7 may be due to the timing differences between replicate handling.

In any case, scientists cannot observe such interesting structures from any models. Hypothetically, these structures could lead to interesting post hoc analyses. For instance, if a similar structure existed in data where the authors had noted an inadvertent experimental or biological discrepancy between those replicates, then a post hoc hypothesis that these genes might respond to that discrepant condition could be generated. We note this would only serve as a hypothesis generator; conventional genetic studies and additional evidence would be needed to confirm any possible role these genes have on this biological activity.

### Assessing DEG calls in scatterplot matrices

**Fig. 9:**
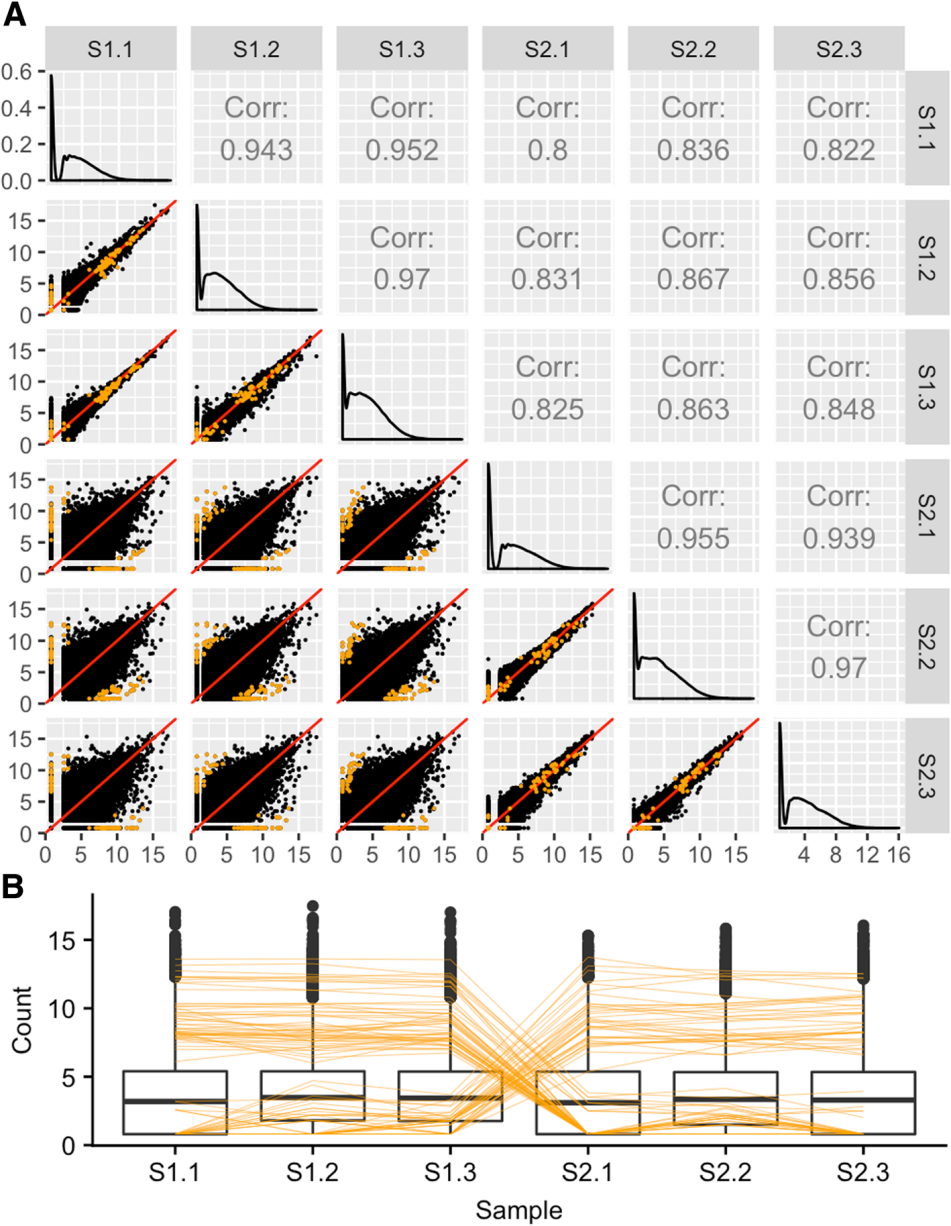
Assessing differential expression in RNA-seq data using scatterplot matrices. Example of the expected structure of DEG calls (in orange) from the soybean cotyledon dataset [27]. In the scatterplot matrix (subplot A), DEGs should fall along the *x=y* line for replicates and deviate from it for treatments. In the parallel coordinate plot (subplot B), DEGs should show levelness between replicates and crosses between treatments. These two plotting types can be linked to quickly provide users multiple perspectives of their DEG calls

The scatterplot matrix can also be used to quickly examine the DEGs returned from a given model. Figure 9 shows the DEGs from the soybean cotyledon dataset superimposed as orange points onto the scatterplot matrix [27]. We expect for DEGs to fall along the *x=y* line for scatterplots between replicates and deviate from the *x=y* line for scatterplots between treatment groups, as is confirmed in Fig. 9. As a side note, we could also link these DEGs as parallel coordinate lines on a side-by-side boxplot to confirm the expected pattern of differential expression from a second viewpoint. If we do not observe what should be expected of DEGs, then the DEG calls from the model may need to be scrutinized further.

## Litre plots

We demonstrated how to view DEGs onto the Cartesian coordinates of the scatterplot matrix in Fig. 9. Unfortunately, this figure becomes limited when we investigate treatment groups that contain a large number of replicates because we then have too many small scatterplots for it to remain an effective visualization tool. Moreover, researchers could benefit from additional plotting tools that allow them to quickly verify individual DEGs returned from a model. As a result, we developed a plot that allows users to visualize *one* DEG of interest onto the Cartesian coordinates of *one* scatterplot matrix.

The “replicate line plot" was developed by researchers who demonstrated it could detect model scaling problems in microarray data [31]. Unfortunately, this plot is only applicable on datasets where treatment groups contain exactly two replicates. The plot we now introduce is an extension of the “replicate line plot" that can be applied to datasets with two or more replicates. We call this new plot a repLIcate TREatment (“litre") plot.

**Fig. 10:**
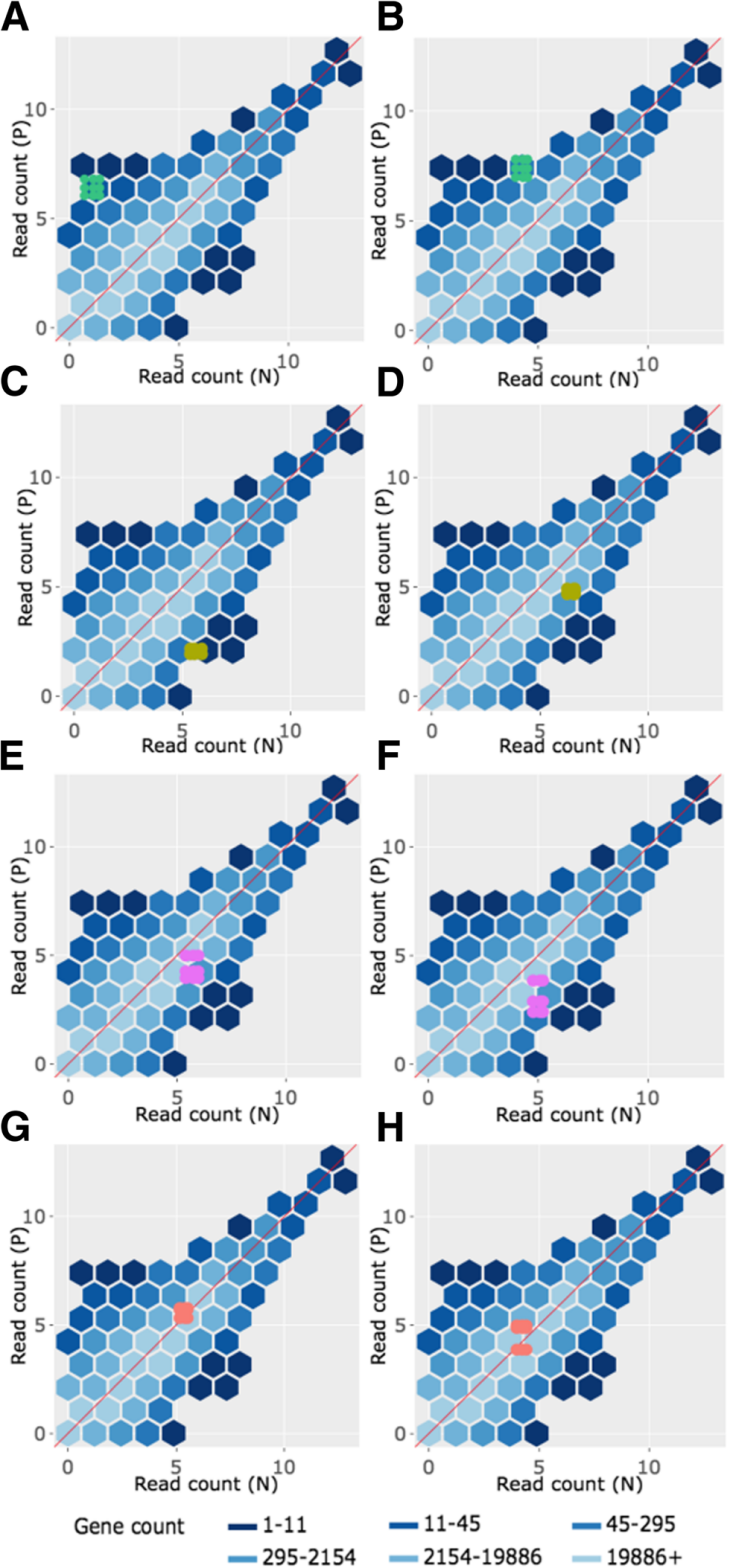
Example litre plots for clustered significant genes in the soybean iron metabolism data. Litre plots for representative genes from clusters created in Fig. 2 [24]. Subplots **a** and **b** each show a gene from Cluster 1 overlaid as green points. Subplots **c** and **d** each show a gene from Cluster 2 overlaid as dark yellow points. Subplots **e** and **f** each show a gene from Cluster 3 overlaid as pink points. Subplots **g** and **h** each show a gene from Cluster 4 overlaid as orange points

In the litre plot, each gene is plotted once for each possible combination of replicates between treatment groups. For example, there are nine ways to pair a replicate from one treatment group with a replicate from the other treatment group in the soybean iron-metabolism dataset (N.1 and P.1, N.1 and P.2, N.1 and P.3, N.2 and P.1, N.2. and P.2, N.2 and P.3, N.3 and P.1, N.3 and P.2, and N.3 and P.3) [24]. Hence, each gene in this dataset is plotted as nine points in the litre plot. With 56,044 genes in this data, we would need to plot 504,396 points. This would reduce the speed of interactive functionality as well as cause overplotting problems. As a result, we again use hexagon bins to summarize this massive information (Fig. 10 shows eight example litre plots).

Once the background of hexagons has been drawn to give us a sense of the distribution of all between-treatment sample pair combinations for all genes, the user can superimpose the nine points of one gene of interest. We can examine and compare litre plots using the clusters we created in Fig. 2. Subplots A and B of Fig. 10 each show a significant gene from Cluster 1 plotted as nine green points, subplots C and D each show a significant gene from Cluster 2 plotted as nine dark yellow points, subplots E and F each show a significant gene from Cluster 3 plotted as nine pink points, and subplots G and H each show a significant genes from Cluster 4 plotted as nine orange points.

For the case of Fig. 10a and b, the nine overlaid points are superimposed in a manner we would expect from a DEG: They are located far from the *x=y* line (difference between treatments) and are close to each other (similarity between replicates). Figure 10c and d also show expected patterns for DEGs, although the genes are now overexpressed in the other treatment (group N). The replicates in subplot D are so precise that the overlaid points almost entirely overlap each other. In contrast, Fig. 10e and f do not seem to show as much replicate consistency. Now, there seems to be a pattern in which one replicate from the P group is larger than (and visually distanced from) the other two replicates. In other words, litre plots are able to capture the pattern differences in the significant genes from Cluster 2 and 3 that we saw back in Fig. 2.

Moreover, in the case of Fig. 10g and h, the nine overlaid points are not clearly superimposed in the distinct pattern we expect of significant genes. While subplot G shows a gene that has consistent replications, the difference between the treatment groups is so small that the overlaid points cluster around the *x=y* line. Additionally, the gene displayed in subplot H shows inconsistent replications and consistent treatment groups, as the spread-out overlaid points center on the *x=y* line. Despite these genes being deemed significant by the model, the litre plots call into question whether the genes from this cluster show an expected profile of differential expression. This is similar to the messy-looking parallel coordinate plots we saw from these genes in Cluster 4 back in Fig. 2. As a result, litre plots can detect odd and questionable patterns in individual “significant genes" that cannot be detected numerically through models. If this happens, the user may wish to further investigate these DEG calls.

Interactive litre plots are available online for the Cluster 1 significant genes (Fig. 10a and b) [32], Cluster 2 significant genes (Fig. 10c and d) [33], Cluster 3 significant genes (Fig. 10e and f) [34], and Cluster 4 significant genes (Fig. 10g and h) [35]. As can be verified in the interactive versions of the litre plot, users are provided several input fields that tailor the plot functionality. For instance, the user can easily select which treatment pair to explore (for data that contains more than two treatment groups) and can quickly scroll through significant genes one by one in order of increasing FDR values. Please read the “About” tab in the interactive links for more information.

**Fig. 11:**
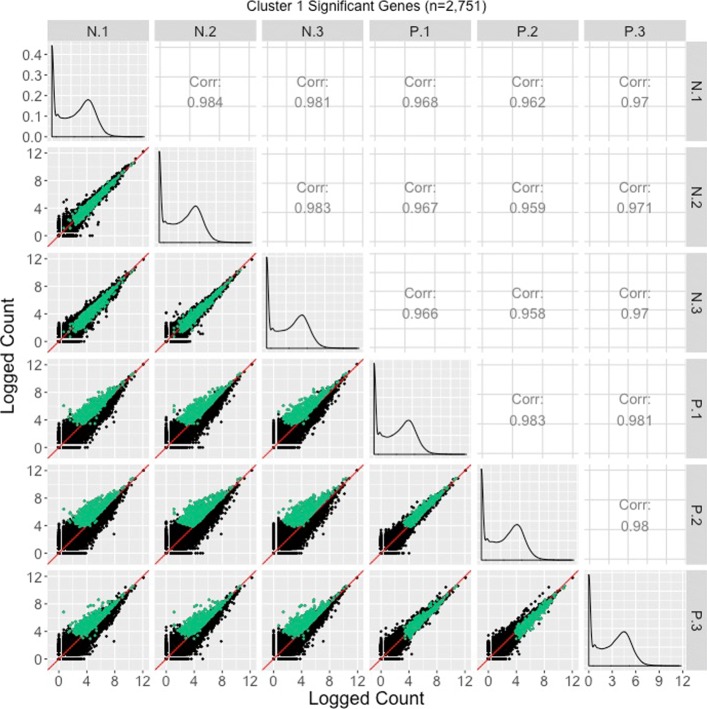
Cluster 1 significant genes from the soybean iron metabolism data overlaid on a scatterplot matrix. Example of using a scatterplot matrix to assess DEG calls from a model in the iron-metabolism soybean dataset. There were 2751 significant genes in Cluster 1 after performing a hierarchical clustering analysis with a cluster size of four (Fig. 2). These significant genes are overlaid in green on the scatterplot matrix. They follow the expected patterns of differential expression with most green points falling along the *x=y* line in the scatterplots between replicates, but deviating from the *x=y* line in the scatterplots between treatments. The deviation consistently demonstrates higher expression in the P group than in the N group. Hence, these green points seem to represent DEGs that were significantly overexpressed in the P group, which draws the same conclusion with what we derived using the parallel coordinate plots in Fig. 2. One difficulty with plotting such a large number of DEGs onto the scatterplot matrix is that overplotting can obscure our inability to determine how many DEGs are in a given location. This is why we should also view these genes individually in litre plots (Fig. 10a and b)

**Fig. 12:**
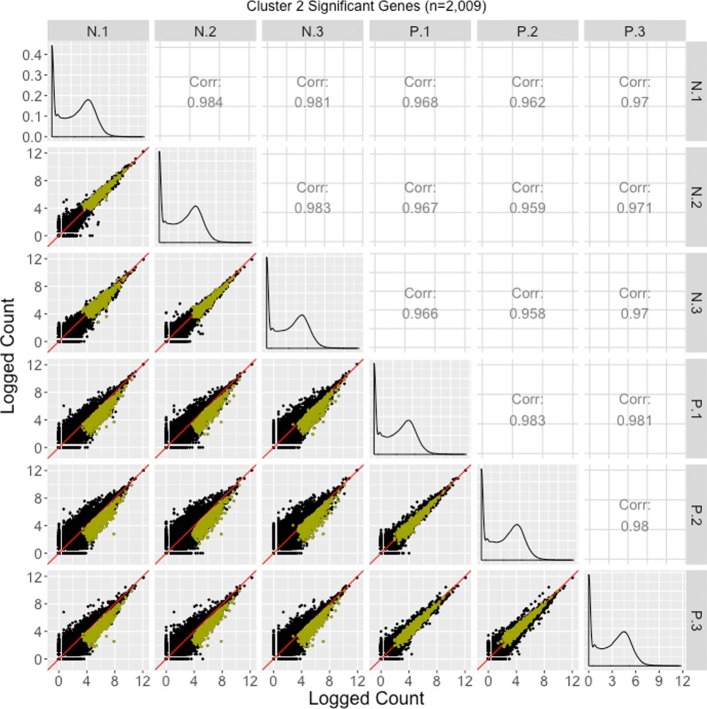
Cluster 2 significant genes from the soybean iron metabolism data overlaid on a scatterplot matrix. Example of using a scatterplot matrix to assess DEG calls from a model in the iron-metabolism soybean dataset. There were 2009 significant genes in Cluster 2 after performing a hierarchical clustering analysis with a cluster size of four (Fig. 2). These significant genes are overlaid in dark yellow on the scatterplot matrix. They follow the expected patterns of differential expression with most dark yellow points falling along the *x=y* line in the scatterplots between replicates, but deviating from the *x=y* line in the scatterplots between treatments. The deviation consistently demonstrates higher expression in the N group than in the P group. Hence, these dark yellow points seem to represent genes that were significantly overexpressed in the N group, which draws the same conclusion with what we derived using the parallel coordinate plots in Fig. 2. One difficulty with plotting such a large number of DEGs onto the scatterplot matrix is that overplotting can obscure our inability to determine how many DEGs are in a given location. This is why we might also view these genes individually in litre plots (Fig. 10c and d)

**Fig. 13:**
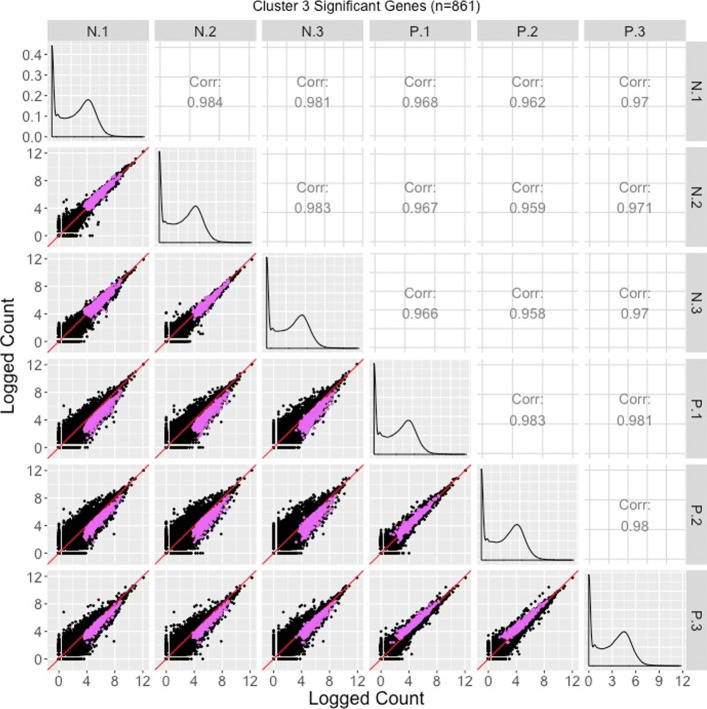
Cluster 3 significant genes from the soybean iron metabolism data overlaid on a scatterplot matrix. Example of using a scatterplot matrix to assess DEG calls from a model in the iron-metabolism soybean dataset. There were 861 significant genes in Cluster 3 after performing a hierarchical clustering analysis with a cluster size of four (Fig. 2). These significant genes are overlaid in pink on the scatterplot matrix. For the most part, they follow the expected patterns of differential expression with pink points falling along the *x=y* line in the scatterplots between replicates, but deviating from the *x=y* line in the scatterplots between treatments. The deviation consistently demonstrates higher expression in the N group than in the P group. The scatterplot between replicates P.1 and P.3 shows slightly higher expression in P.3, and the scatterplot between replicates P.2 and P.3 also shows slightly higher expression in P.3. Hence, these pink points seem to represent genes that were significantly overexpressed in the N group, but with slight inconstencies in the replicates in the P group, which matches what we saw in the parallel coordinate plots in Fig. 2. One difficulty with plotting such a large number of DEGs onto the scatterplot matrix is that overplotting can obscure our inability to determine how many DEGs are in a given location. This is why we might also view these genes individually in litre plots (Fig. 10e and f)

**Fig. 14:**
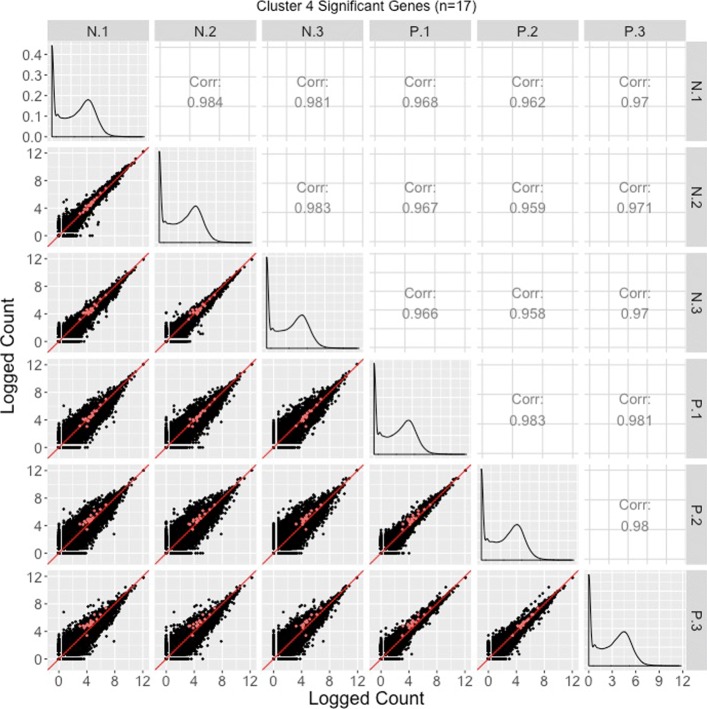
Cluster 4 significant genes from the soybean iron metabolism data overlaid on a scatterplot matrix. Example of using a scatterplot matrix to assess DEG calls from a model in the iron-metabolism soybean dataset. There were 17 significant genes in Cluster 4 after performing a hierarchical clustering analysis with a cluster size of four (Fig. 2). These significant genes are overlaid in orange on the scatterplot matrix. For the most part, they do not seem to follow the expected patterns of differential expression: In many of the scatterplots between treatments, the orange points do not seem to deviate much from the *x=y* line. Moreover, in the scatterplots between P.1 and P.2 as well as P.1 and P.3, the orange points seems to indicate an underexpression of the P.1 replicate. We similarly saw somewhat messy looking DEG calls in Cluster 4 in the form of parallel coordinate plots (Fig. 2) and litre plots (Fig. 10g and h)

Corresponding scatterplot matrices with the DEGs from these four clusters overlaid can be viewed in Figs. 11, 12, 13, 14. Readers can verify that the parallel coordinate plots, litre plots, and scatterplot matrices tell a similar story about the DEG patterns in these four clusters.

## Closing case study

We briefly discuss an additional example that merges many of the topics addressed in this paper. The publicly available data for this example contain technical replicates of liver and kidney RNA samples from one human male [12]. We first calculate DEG calls for this data using the normalization method of library size scaling, where the number of total reads in each sample are normalized to a common value across all samples. This process leads to 9018 DEGs, with most of them (∼78%) showing higher expression in the kidney group.

**Fig. 15:**
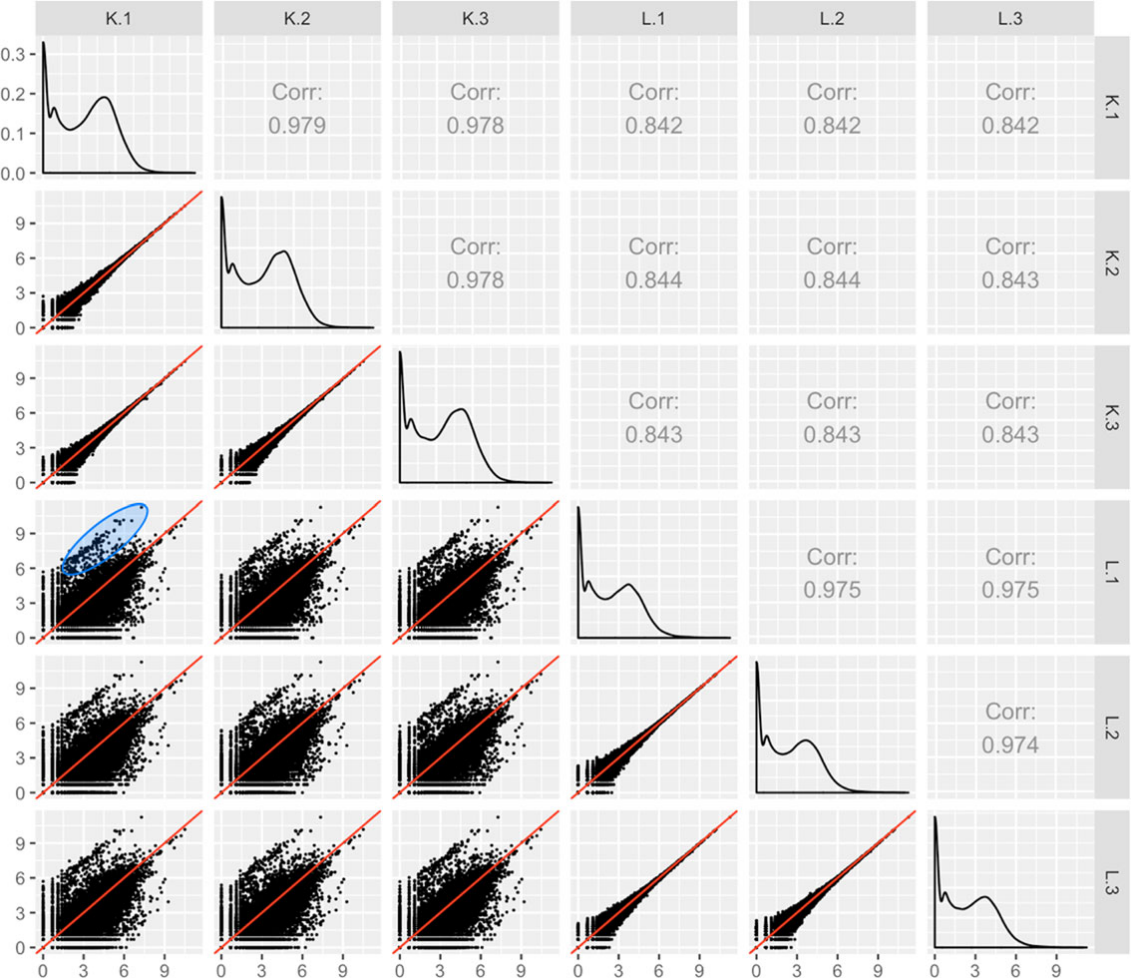
Scatterplot matrix detects unexpected structure in liver and kidney technical replicate RNA-seq dataset. Scatterplot matrix of liver and kidney technical replicates [12]. The technical replicate scatterplots look precise as is expected, with little variability around the *x=y* line. The treatment group scatterplots have much more variability around the *x=y* line, as we would expect. However, each treatment group scatterplot contains a pronounced streak of highly-expressed liver-specific genes, which deviates from the expected distribution (shown in blue oval in one example scatterplot). Some researchers have suggested that differences in the distribution of reads between groups may require particularly stringent normalization

**Fig. 16:**
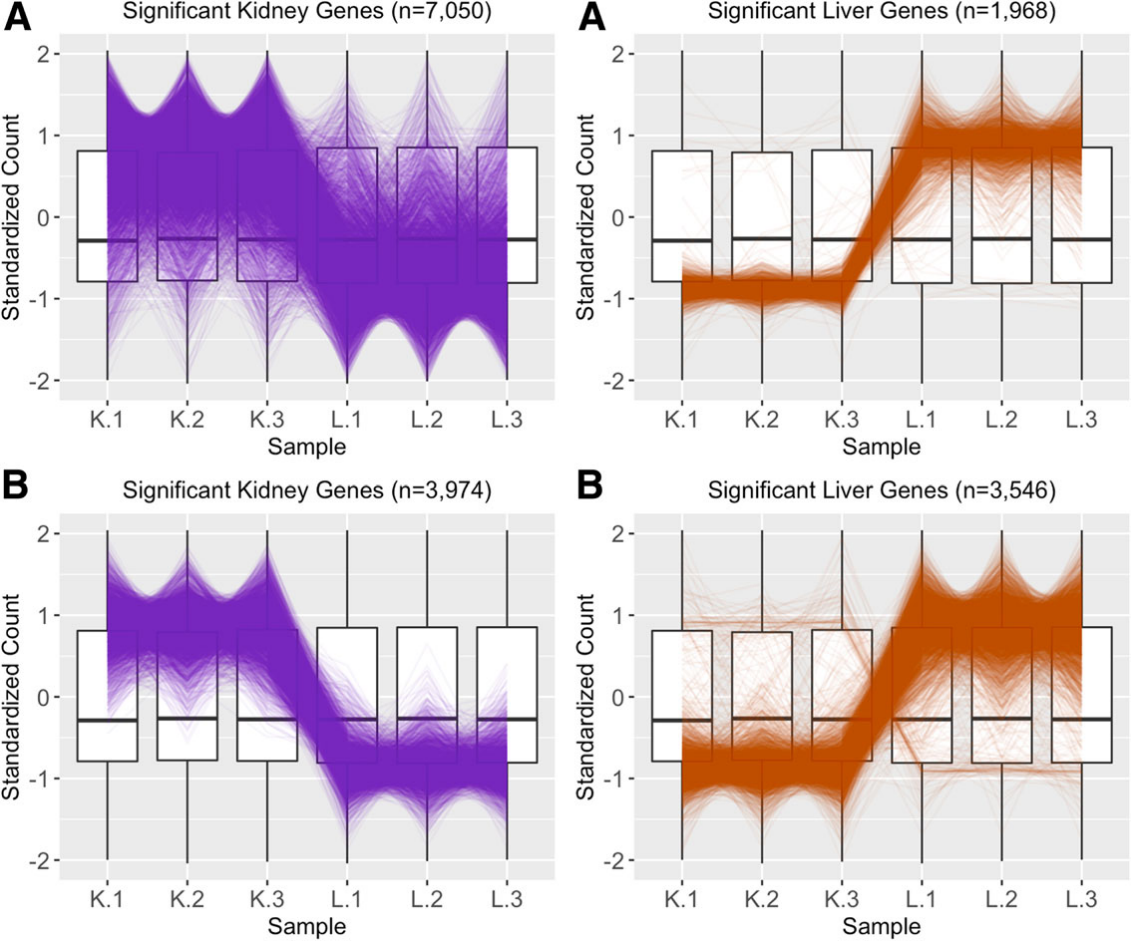
Comparing normalization method effect on significance designation using parallel coordinate plots. Subplot **a** shows parallel coordinate plots of the DEGs from liver and kidney technical replicates [12] after library size scale normalization. The division of DEGs between the two groups was rather disparate, with ∼78% of the DEGs being kidney-specific and only ∼22% of the DEGs being liver-specific. Also of note, while the parallel coordinate patterns of the liver-specific DEGs appear as expected, the patterns of the kidney-specific DEGs seem to show comparatively larger variability between the replicates. Subplot **b** shows parallel coordinate plots of the DEGs from liver and kidney technical replicates after TMM normalization. The division of DEGs between the two groups is more balanced than in Subplot **a**, with ∼53% of the DEGs being kidney-specific and ∼47% of the DEGs being liver-specific. Additionally, the parallel coordinate patterns of the kidney DEGs is vastly improved. However, the parallel coordinate patterns of the liver DEGs is slightly more messy looking. As a result, we investigate the effects of normalization on this data more thoroughly

Although we could finish our analysis at this point and draw conclusions based on this list of DEGs that came from the model, it would be wise to also visualize this dataset. Viewing this data as a scatterplot matrix confirms the expected pattern with treatment scatterplots showing larger variation than technical replicate scatterplots (Fig. 15). However, it also uncovers a hidden pattern in the treatment plots: There is a pronounced streak of genes with higher expressions in the liver group (highlighted with a blue oval in Fig. 15). We should also view the DEGs from the model using parallel coordinate plots: Upon doing so, we notice that while the 1968 liver-specific DEGs follow the expected pattern of significant calls, a substantial fraction of the 7050 kidney-specific DEGs appear comparatively noisy (Fig. 16a).

Taking both of these observations into account, we may need to reconsider our normalization technique. Some authors have argued that library size scaling method is not adequate in all cases, especially when the underlying distribution of reads between samples is inconsistent. In the current data, the observed streak of outlier genes that are highly expressed in the liver samples (Fig. 15) reduces the sequencing quota available to the remaning genes in these samples, which could create an articial inflation of the kidney-specific DEG calls. These authors have recommended trimmed mean of M values (TMM) normalization for such cases (including for this particular dataset) as this technique generates sample scaling factors that consider sample distributions [15].

In light of all this, we re-start the analysis and now apply TMM normalization to this data. This process leads to 7520 DEGs that have a more level distribution between the kidney (∼53%) and liver (∼47%) groups. The scatterplot matrix did not appear differently from what we saw in Fig. 15 as both of these normalization methods are scaling procedures. However, we should visualize the new DEG calls. Plotting these DEGs as parallel coordinate lines paints a much cleaner picture from what we saw earlier, with most genes following the expected pattern of significance (Fig. 16b). Of the 7050 kidney-specific DEGs we saw previously with library size scaling normalization, only a much cleaner-looking subset (*n*=3974) of them remained as such using TMM normalization. TMM normalization kept the original 1968 liver-specific DEGs from library size scaling but added 1578 more for a total of 3546 liver-specific DEGs. As such, it appears that the liver-specific DEGs may be slightly less clean-looking with TMM normalization. We emphasize that the 3974 kidney-specific DEGs from TMM normalization are a proper subset of the 7050 kidney-specific DEGs from library scale normalization, and the 1968 liver-specific DEGs from library scale normalization are a proper subset of the 3546 liver-specific DEGs from TMM normalization.

We therefore perform a deeper investigation of the effects of normalization on this data. To do this, we thoroughly explore four subsets of genes from this case study in the form of parallel coordinate plots, scatterplot matrices, and litre plots. We also demonstrate the use of data standardization for scatterplot matrices and litre plots as a means to magnify certain informative patterns. In this thorough examination, we will use consistent color-coding when plotting example genes from each of the four gene subsets. The four gene subsets and their color-codes are as follows:

1. The 3974 kidney-specific DEGs from library size scale normalization that remained as DEGs even after TMM normalization. These DEGs will be plotted in purple. As these genes were declared significant with both library size scale normalization and TMM normalization, we expect them to follow the expected patterns of DEGs.
2. The 1968 liver-specific DEGs from library size scale normalization that remained as DEGs even after TMM normalization. These DEGs will be plotted in orange. As these genes were declared significant with both library size scale normalization and TMM normalization, we expect them to follow the expected patterns of DEGs.
3. The 3076 kidney-specific DEGs from library size scale normalization that were *removed* as DEGs using TMM normalization. These DEGs will be plotted in red. As these genes were removed from DEG designation with the more-appropriate TMM normalization, we expect them to *not* convincingly follow the expected patterns of DEGs.
4. The 1578 liver-specific genes that were not detected as DEGs with library size scale normalization but were then *added* as such using TMM normalization. These DEGs will be plotted in pink. As these genes were not declared significant with library size scale normalization but were then declared as significant using the more-appropriate TMM normalization, we expect them to *somewhat* convincingly follow the expected patterns of DEGs.

**Fig. 17:**
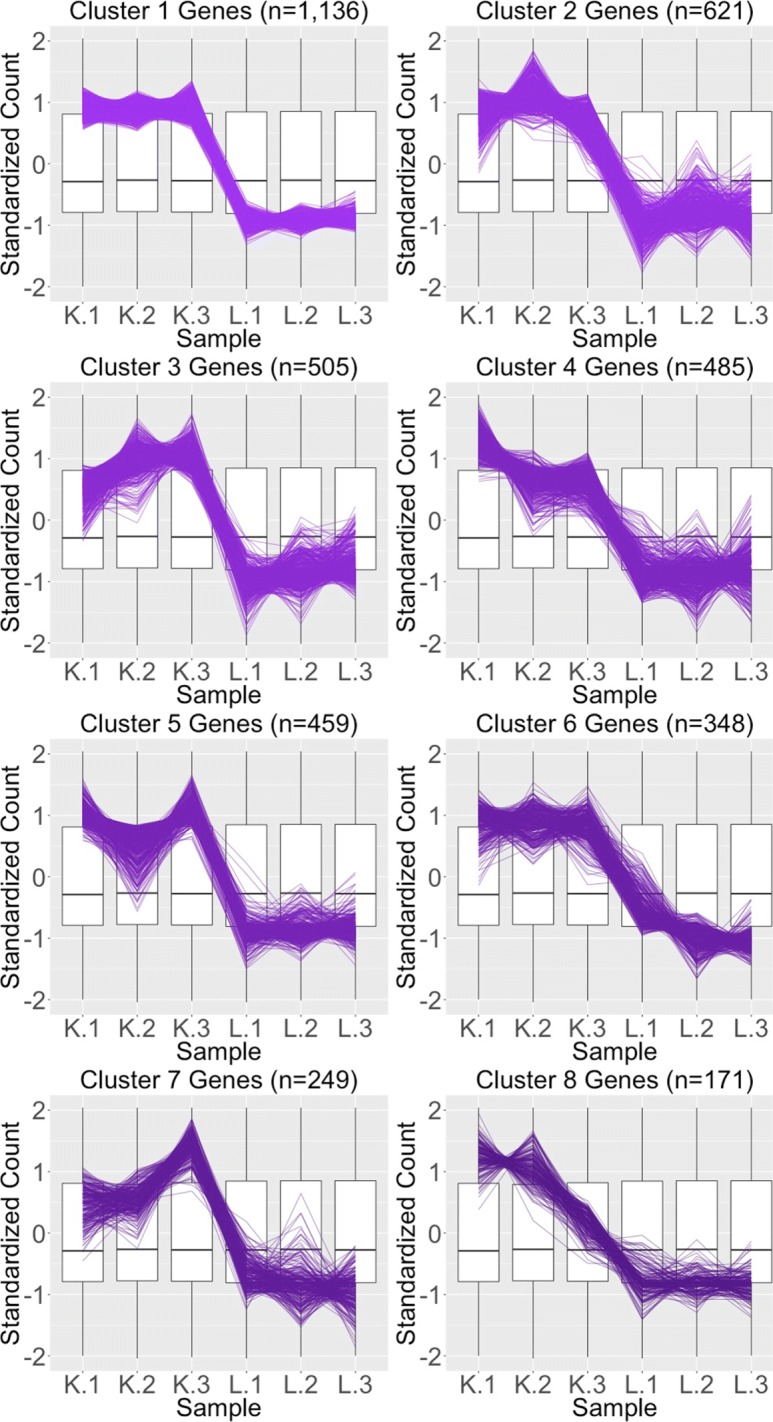
Parallel coordinate plots for gene clusters that remained as kidney-specific DEGs after TMM normalization. Parallel coordinate plots showing eight hierarchical clusters from the 3974 genes that remained in the kidney-specific DEGs after TMM normalization. We see that, for the most part, the parallel coordinate patterns follow the expected patterns across the clusters. The ideal pattern of DEGs is especially captured in the first cluster (the largest one with 1136 genes). We applied ombre coloring across the clusters in order of cluster size. We used hierarchical clustering to tease apart subtle pattern differences and to mitigate additional overplotting that would occur if we were to plot all genes onto only one parallel coordinate plot. The side-by-side boxplots represent *all* gene counts in the dataset

**Fig. 18:**
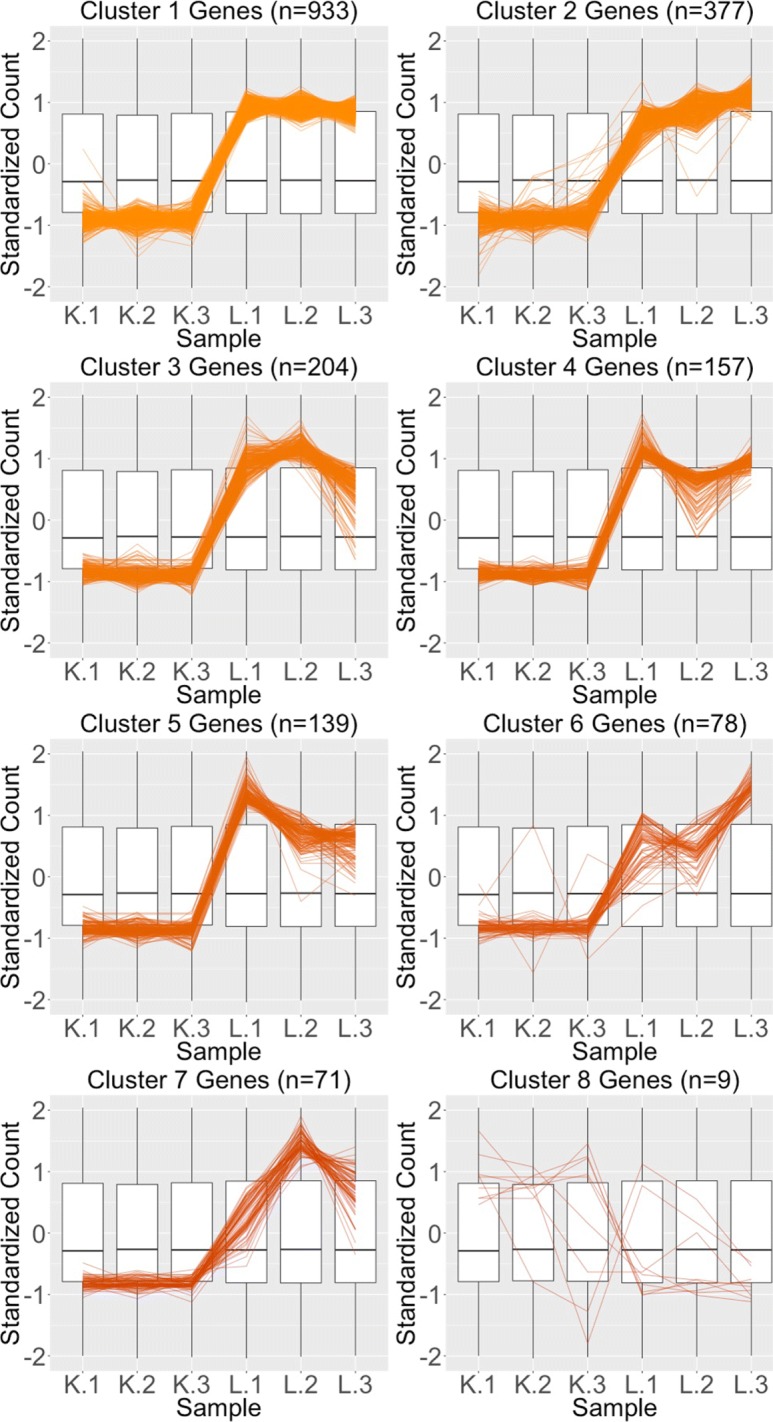
Parallel coordinate plots for gene clusters that remained as liver-specific DEGs after TMM normalization. Parallel coordinate plots showing eight hierarchical clusters from the 1968 genes that remained in the liver-specific DEGs after TMM normalization. We see that, for the most part, the parallel coordinate patterns follow the expected patterns across the clusters. The ideal pattern of DEGs is especially captured in the first cluster (the largest one with 933 genes). We applied ombre coloring across the clusters in order of cluster size. We used hierarchical clustering to tease apart subtle pattern differences and to mitigate additional overplotting that would occur if we were to plot all genes onto only one parallel coordinate plot. The side-by-side boxplots represent *all* gene counts in the dataset

**Fig. 19:**
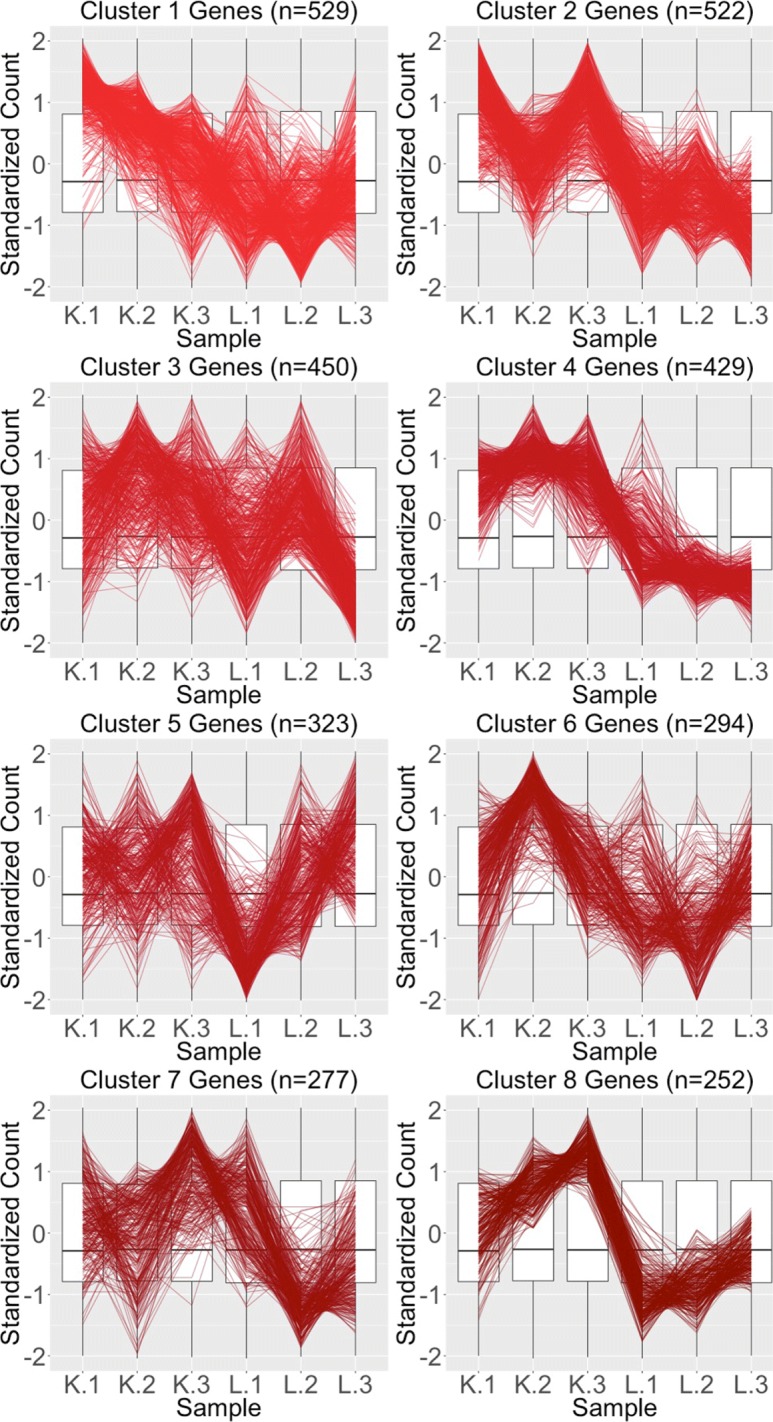
Parallel coordinate plots for gene clusters that were removed from kidney-specific DEGs after TMM normalization. Parallel coordinate plots showing eight hierarchical clusters from the 3076 genes that were removed from the kidney-specific DEGs after TMM normalization. The patterns in almost all clusters do not resemble the expected DEG format; instead, they show large variability between replicates and small variability between treatments. This plot provides additional statistical evidence that the application of TMM normalization successfully removed genes that were previously mislabeled as kidney-specific DEGs with library size scaling normalization. We used hierarchical clustering to tease apart subtle pattern differences and to mitigate overplotting. The side-by-side boxplots represent *all* gene counts in the dataset

**Fig. 20:**
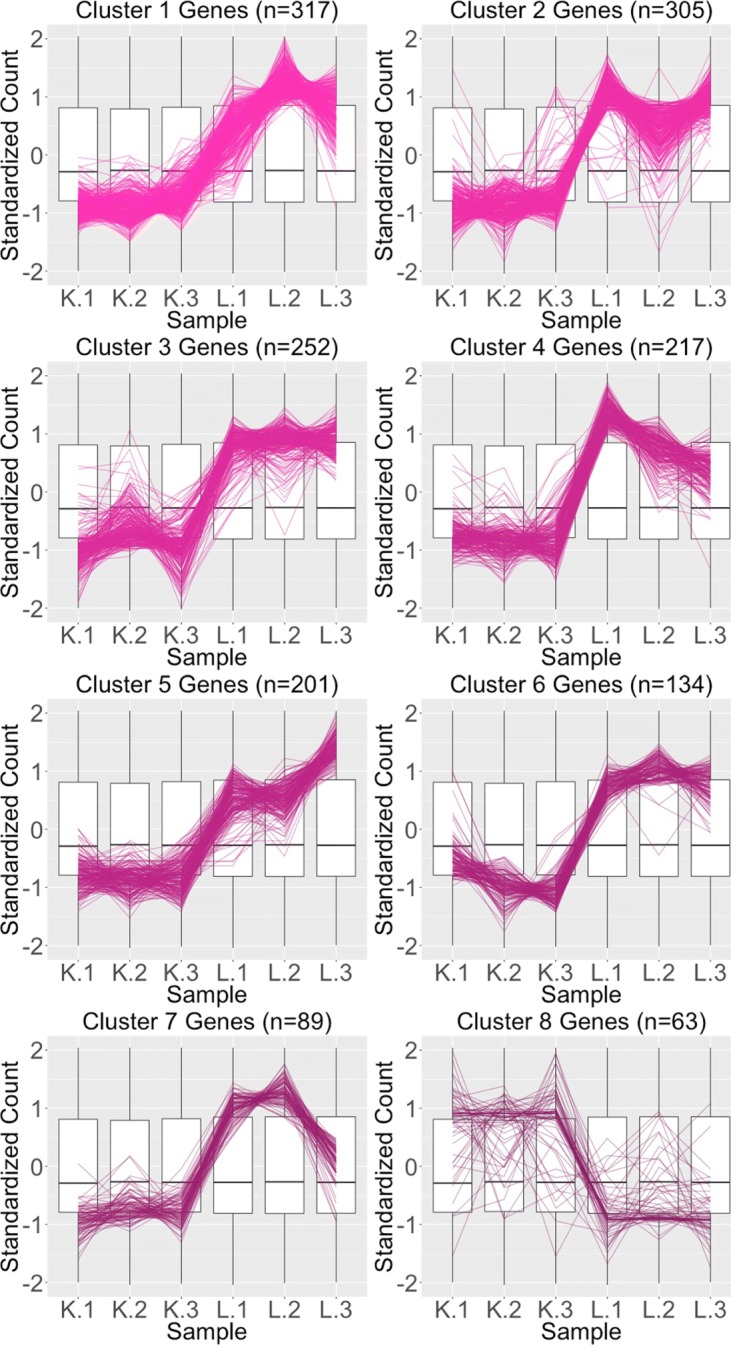
Parallel coordinate plots for gene clusters that were added as liver-specific DEGs after TMM normalization. Parallel coordinate plots showing eight hierarchical clusters from the 1578 genes that were *added* as liver-specific DEGs after TMM normalization. We see that the parallel coordinate lines *somewhat* follow the expected patterns across the clusters, better than what we saw in the red (Fig. 19) gene subsets, but not as precisely as we saw with the purple (Fig. 17) and orange (Fig. 18) gene subsets. We applied ombre coloring across the clusters in order of cluster size. We used hierarchical clustering to tease apart subtle pattern differences and to mitigate additional overplotting that would occur if we were to plot all genes onto only one parallel coordinate plot. The side-by-side boxplots represent *all* gene counts in the dataset

We begin by plotting the four gene subsets in the form of parallel coordinate plots after application of hierarchical clustering analysis (Figs. 17,18, 19 through 20). Each subset is grouped into eight clusters, not only to separate the genes into any subtle pattern differences, but also to reduce any overplotting that would occur should they all be viewed together as one large cluster. Figures 17 and 18 show that the genes designated as DEGs in both forms of normalization (purple and orange) have clean-looking patterns (especially in their largest cluster), Fig. 19 shows that the genes removed with TMM normalization (red) have messy-looking parallel coordinate plots, and Fig. 20 shows that the genes added with TMM normalization (pink) have parallel coordinate plots that are less clean than those in Figs. 17 and 18 but more clean than those in Fig. 19.

**Fig. 21:**
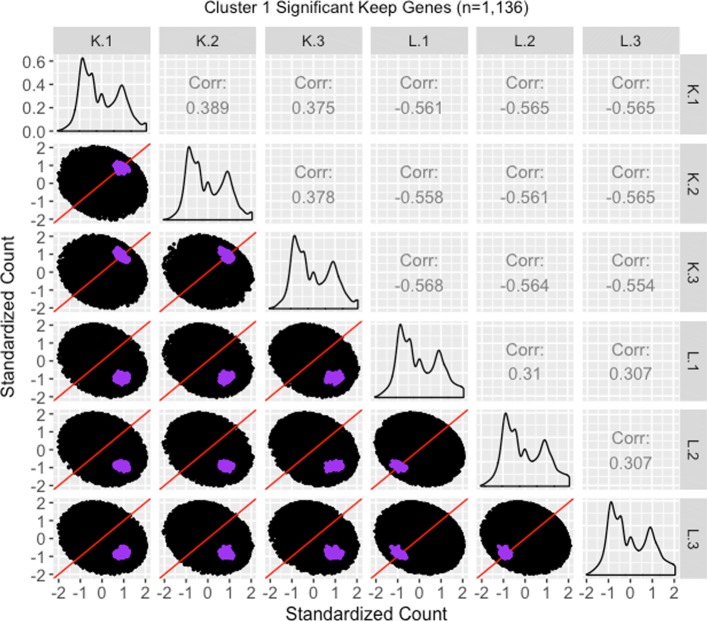
Standardized scatterplot matrix for gene cluster that remained as kidney-specific DEGs after TMM normalization. Scatterplot matrix of the *standardized* 1136 genes that were in the first cluster (Fig. 17) from genes that remained as kidney-specific DEGs even after TMM normalization. Even though the standardization process removes the interesting geometrical features we would otherwise see, it amplifies DEG patterns more clearly. Here, the highlighted genes appear more clustered and separated from the *x=y* line in the treatment scatterplots, and more clustered and connected to the *x=y* line in the replicate scatterplots. We can also now see more clearly in the replicate scatterplots that the kidney expression is higher than the liver expression

**Fig. 22:**
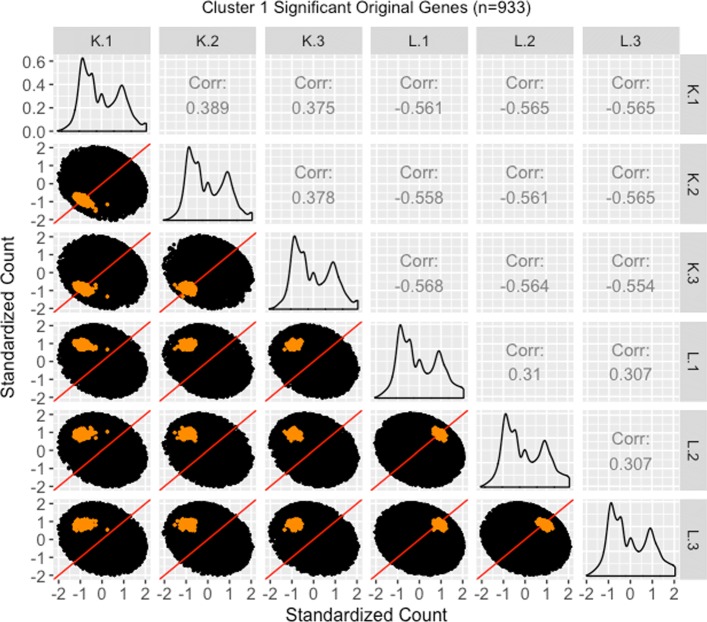
Standardized scatterplot matrix for gene cluster that remained as liver-specific DEGs after TMM normalization. Scatterplot matrix of the *standardized* 933 genes that were in the first cluster (Fig. 18) from genes that remained as liver-specific DEGs even after TMM normalization. Even though the standardization process removes the interesting geometrical features we would otherwise see, it amplifies DEG patterns in meaningful ways. Here, the highlighted genes appear more clustered and separated from the *x=y* line in the treatment scatterplots, and more clustered and connected to the *x=y* line in the replicate scatterplots. We can also now see more clearly in the replicate scatterplots that the liver expression is higher than the kidney expression

**Fig. 23:**
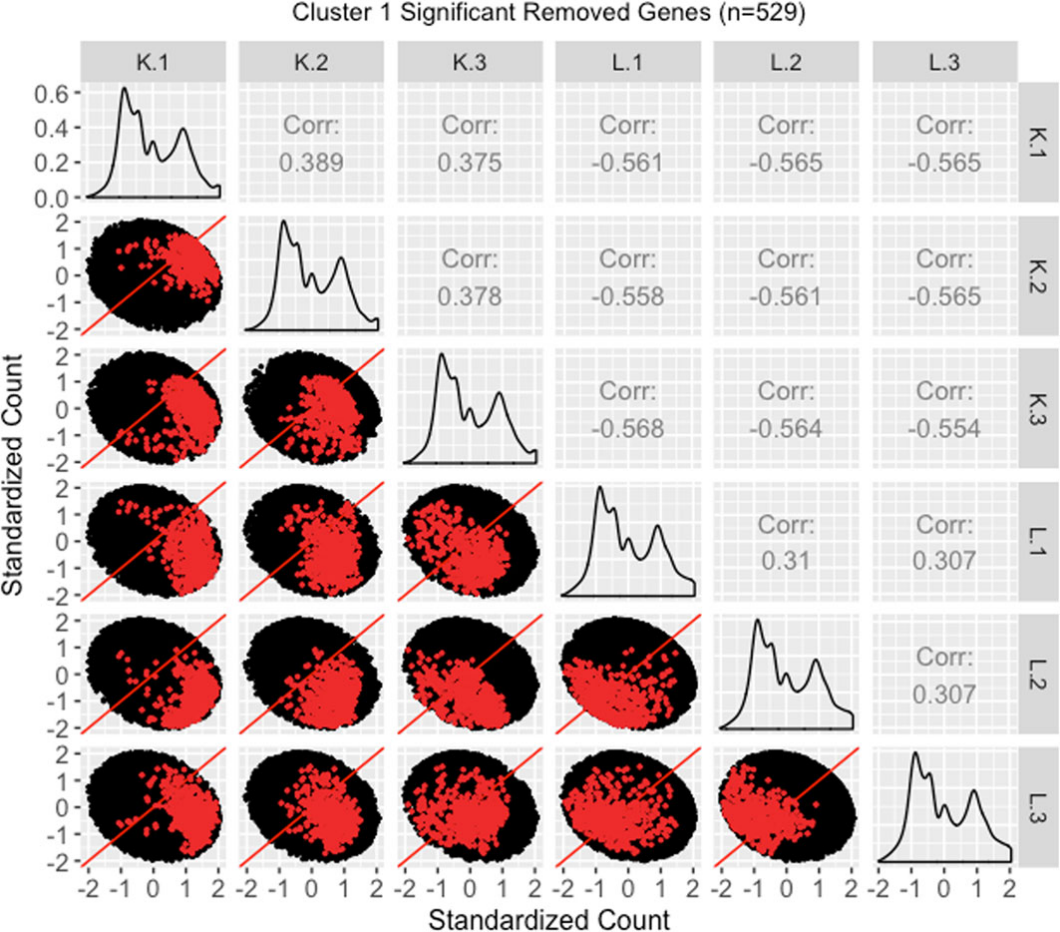
Standardized scatterplot matrix for gene cluster that were removed from kidney-specific DEGs after TMM normalization. Scatterplot matrix of the *standardized* 529 genes that were in the first cluster (Fig. 19) from genes that no longer remained as kidney-specific DEGs after TMM normalization. Even though the standardization process removes the interesting geometrical features we would otherwise see, it amplifies DEG patterns in meaninful ways. Namely, the genes of interest are now spread out more, and the replicate and treatment scatterplots are almost indistinguishable from each other, with both of them showing genes of interest crossing both sides of the *x=y* line. In other words, standardization of the data provides clear visualization evidence that TMM normalization was justified in removing these genes from DEG designation

We continue our visualization study by overlaying genes from the largest cluster of the four gene subsets in the form of *standardized* scatterplot matrices (Figs. 21 22, 23 through 24). Notice that standardization causes the whole dataset to appear as oval-shapes that are almost identical across all scatterplots. In other words, when we standardize our scatterplot matrices, we lose geometric structures that can elicit meaningful information about the dataset as a whole like we saw in Figs. 3, 4, 5, 7, 9, and 15. However, in compensation for losing useful information about the whole dataset, standardization often amplifies meaningful patterns in the overlaid DEGs. Should the reader be interested, Additional files 1, 2, 3, 4 show the same scatterplot matrices as the current case study below (Figs. 21, 22, 23 through 24) only not standardized. The reader can verify that the overlaid DEG patterns are more spread out in the standardized version, allowing for better interpretation.

**Fig. 24:**
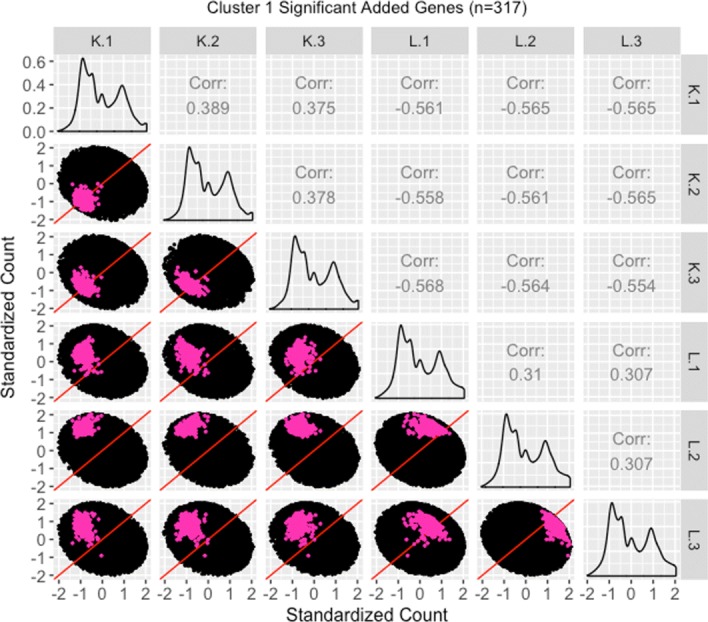
Standardized scatterplot matrix for gene cluster that were added as liver-specific DEGs after TMM normalization. Scatterplot matrix of the *standardized* 317 genes that were in the first cluster (Fig. 20) from genes that were *added* as liver-specific DEGs after TMM normalization. Even though the standardization process removes the interesting geometrical features we would otherwise see, it amplifies DEG patterns in meaningful ways. Namely, the genes of interest are now spread out more, and we can now distinguish the replicate and treatment scatterplots more clearly. For the most part, the genes of interest deviate from the *x=y* line in the treatment scatterplots more so than in the replicate scatterplots, and hence display somewhat of the pattern of differential expression. In fact, the pink genes again appear as an intermediate between the purple and orange genes that clearly display differential expression (Figs. 21 and 22) and the red genes that clearly do *not* display differential expression (Fig. 23). In other words, standardized scatterplot matrices provide additional visualization evidence that TMM normalization was justified in removing the red genes from and adding the pink genes to DEG designation

In general, we see that the genes that were called DEGs in both forms of normalization (purple and orange) have the expected differential expression profiles in the standardized scatterplot matrices, deviating from the *x=y* line in the treatment scatterplots in the anticipated direction (Figs. 21 and 22). The standardized red gene profiles show widely dispersed genes that sometimes deviate from the *x=y* line in the replicate scatterplots and cross both sides of and sometimes stick to the *x=y* line in the treatment scatterplots (Fig. 23). In other words, the red gene profiles often show patterns not akin to differential expression, which we would expect from genes that were *removed* as DEGs with TMM normalization. In contrast, the standardized pink gene profiles show less-widely dispersed genes that deviate less from the *x=y* line in the replicate scatterplots and deviate more from the *x=y* line in the treatment scatterplots (Fig. 24). In other words, the pink gene profiles show patterns more akin to differential expression than the red genes, which we would expect from genes that were *added* as DEGs with TMM normalization. At the same time, the pink gene profiles are not as clean-looking as the purple and orange genes that were designated as DEGs in both forms of normalization. Overall, in these standardized scatterplot matrices, the pink genes appear as an intermediate between the clean-looking purple and orange genes and the messy-looking red genes, which we might expect.

**Fig. 25:**
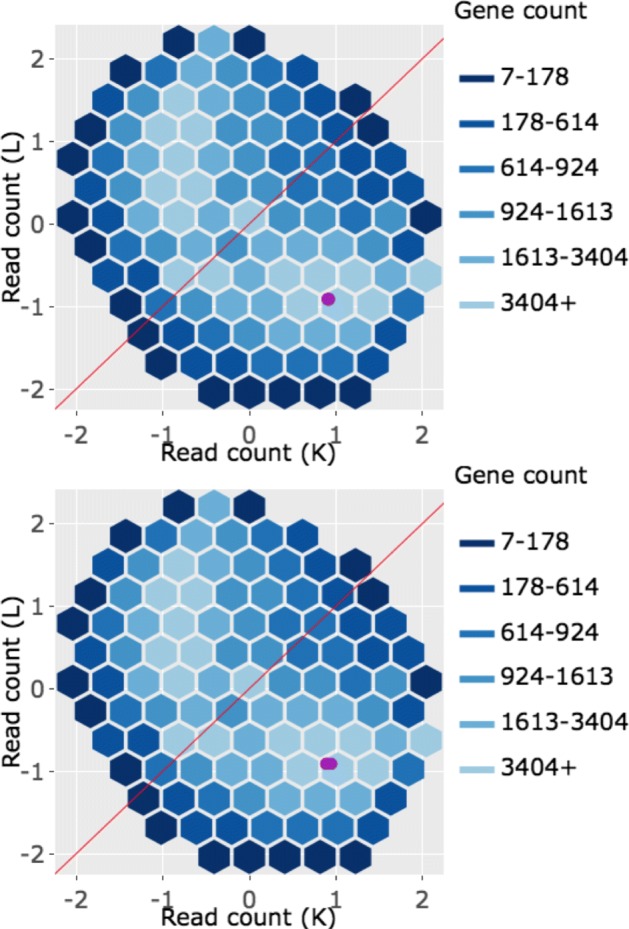
Example standardized litre plots for genes that remained as kidney-specific DEGs after TMM normalization. Example *standardized* litre plots from the 1136 genes that were in the first cluster (Fig. 17) of genes that remained as kidney-specific DEGs even after TMM normalization. With standardization, we immediately note that meaningful information about the dataset as a whole (variation between treatments and replicates, normalization, sample mislabeling, and unexpected patterns like streaks) is now gone. In any case, we confirm that these standardized litre plots corroborate that these purple genes demonstrate the expected patterns of DEGs

**Fig. 26:**
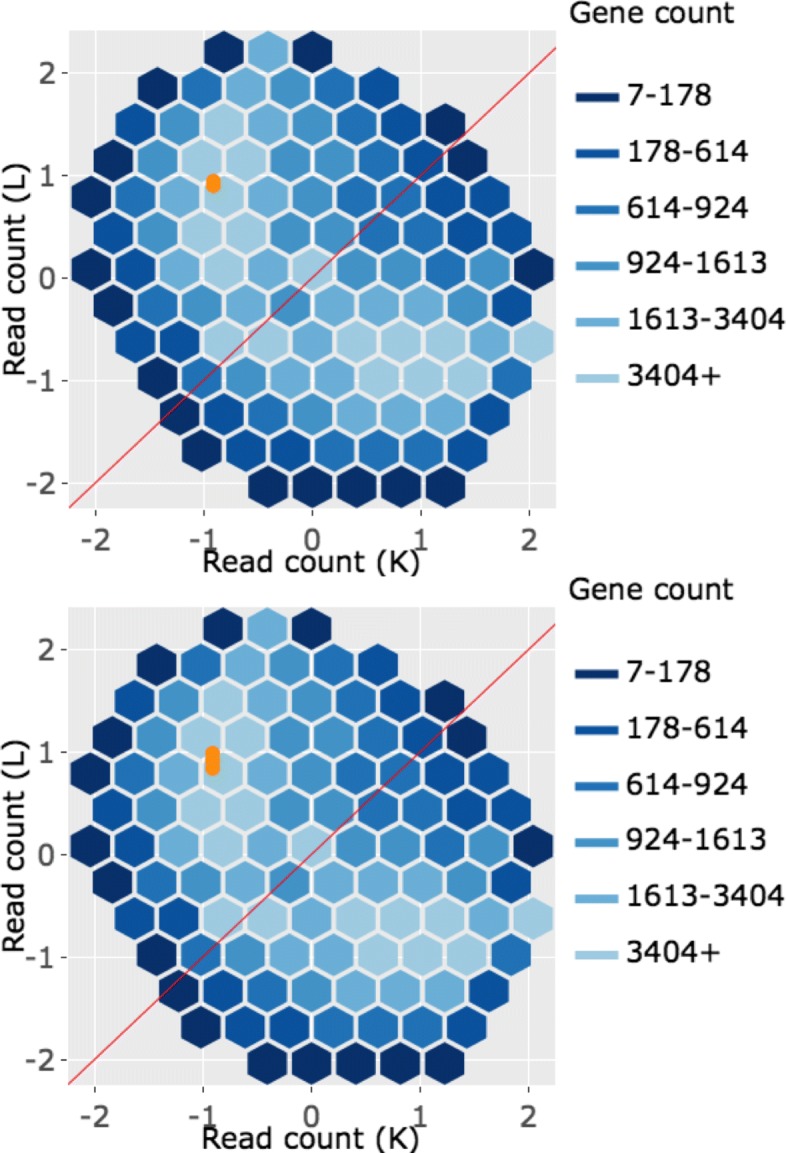
Example standardized litre plots for genes that remained as liver-specific DEGs after TMM normalization. Example litre plots from the 933 genes that were in the first cluster (Fig. 18) from genes that remained as liver-specific DEGs even after TMM normalization. With standardization, we immediately note that meaningful information about the dataset as a whole (variation between treatments and replicates, normalization, sample mislabeling, and unexpected patterns like streaks) is now gone. In any case, we confirm that these standardized litre plots corroborate that these orange genes demonstrate the expected patterns of DEGs

**Fig. 27:**
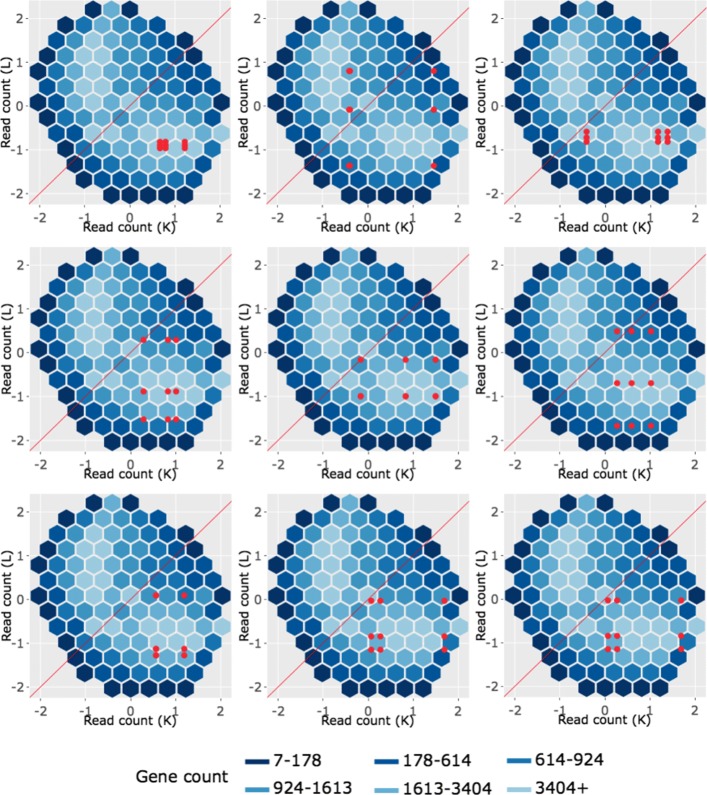
Example standardized litre plots for genes that were removed from kidney-specific DEGs after TMM normalization. *Standardized* litre plots for the nine genes with the lowest FDR values out of the 529 genes that were in the first cluster (Fig. 19) of genes that no longer remained as kidney-specific DEGs after TMM normalization. We verify from an additional perspective that the red genes do not demonstrate the expected patterns of DEGs. The example red genes here are show much larger inconsistencies between replicates than what we saw with the purple (Fig. 25) and orange (Fig. 26) genes. This provides additional evidence that TMM normalization removing these genes from DEG status may be valid

We end our investigation by overlaying example genes from the largest cluster of the four gene subsets in the form of *standardized* litre plots (Figs. 25, 26, 27 through 28). Similar to what we saw earlier, standardization causes the dataset to appear as an oval-shape and removes the original geometric structure in the hexagonal binning. Should the reader be interested, Additional files 5, 6, 7, 8 show the same litre plots as the current case study below (Figs. 25, 26, 27 through 28) only not standardized. The reader can verify that the overlaid DEG patterns are more spread out in the standardized version in the current case study below, allowing for better interpretation.

**Fig. 28:**
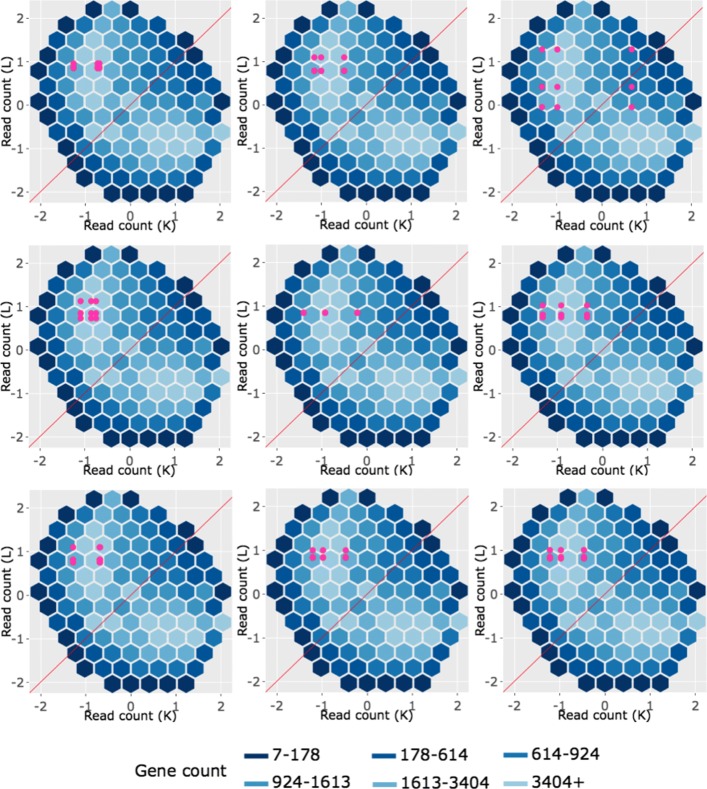
Example standardized litre plots for genes that were added as liver-specific DEGs after TMM normalization. *Standardized* litre plots for the nine genes with the lowest FDR values out of the 317 genes that were in the first cluster (Fig. 20) from genes that were *added* as liver-specific DEGs after TMM normalization. We can quickly determine that the pink profiles in this figure show patterns more akin to differential expression than the red profiles in Fig. 27. That is, the overlaid pink points deviate more from the *x=y* line in a tight cluster than the overlaid red points. At the same time, the overlaid pink points here show patterns less akin to differential expression than the purple (Fig. 25) and orange (Fig. 26) points. In sum, the standardized litre plots again place the pink gene profiles as an intermediate

Overall, we see that the example genes that were called DEGs in both forms of normalization (purple and orange) have the expected profiles in the litre plots, deviating as concentrated bundles away from the *x=y* line (Figs. 25 and 26). The standardized litre plots for the nine genes with the lowest FDR values for both the red (Fig. 27) and pink (Fig. 28) groups allow us to quickly determine that the pink profiles show patterns more akin to differential expression than the red groups. Namely, the overlaid pink points deviate more from the *x=y* line in a tight cluster than the overlaid red points. At the same time, the overlaid pink points show patterns less akin to differential expression than the purple and orange points. All together, the pink gene profiles again appear as intermediates between the clean-looking purple and orange genes and the messy-looking red genes in the standardized litre plots, which is to be expected if TMM normalization is the more appropriate technique.

Our in-depth analyses in this case study collectively suggest that this datasest indeed requires more than just library size scaling for reliable analysis. This case study was meant to underscore the overarching theme of this paper that iteration between models and visualizations is crucial to achieve the most convincing results and conclusions in RNA-seq studies.

## Plot scalability

All visualization plots discussed in this paper have limitations based on the number of samples in the data. Plots that appear messy, regardless of sample numbers, indicate the presence of data quality problems. In general, MDS plots, boxplots, and parallel coordinate plots can remain effective with fairly large sample numbers, particularly if one switches to an aggregate plot rather than points. We note that parallel coordinate plots should be sorted with some metric to help place similar variables near each other, especially when scaling to larger data sets.

Scatterplot matrices usually lose their efficiency at smaller sample numbers due to restricted space: *n*^2^ scatterplots must be drawn for *n*-dimensional data, where *n* is the number of total samples. One remedy is for users to subset their data and plot several smaller scatterplot matrices. We used this technique in our recent honey bee RNA-seq paper where we investigated 2 groups of 12 replicates (24 samples total) [36]. Plotting all samples onto one scatterplot matrix would have required a prohibitive 24×24=576 scatterplots. Instead, we divided the data into four subsets, each with 2 groups of 3 replicates (6 samples total) so that each scatterplot matrix only required 6×6=36 scatterplots. See additional files 11-14 of [36].

Litre plots are another remedy for large data sets and can often accommodate more samples than scatterplot matrices. Indeed, in our honey bee RNA-seq paper, we successfully applied litre plots to our full data that contained 2 groups of 12 replicates (24 samples total). In cases where there are two treatment groups with an equal number of replicates, the litre plot draws *n*^2^ number of points, where *n* is the number of replicates in each group. Hence, we were able to draw 12×12=144 dots on the litre plot successfully in our previous paper. See additional file 4 of [36]. We note that the litre plot is more suitable for large datasets than the replicate line plot, which is ideal for 2 groups of 2 replicates (4 samples total).

## Discussion

In this paper, we strived to convince readers that effective visualization should be a crucial part of two-group differential expression analysis. We used real data to demonstrate that scatterplot matrices, parallel coordinate plots, and litre plots help users check for normalization problems, catch common errors in analysis pipelines, and confirm that the variation between replicates and treatments is as expected. We also showed that these graphical tools allow researchers to quickly explore DEG lists that come out of models and ensure which ones make sense from an additional and arguably more intuitive vantage point. Moreover, we demonstrated that our plotting tools allow researchers to discover genes of interest through visual geometric patterns that would otherwise remain undiscovered with models.

In general, scientists might uncover surprising patterns lurking in their data with plots in ways that cannot be achieved with any formulas or models. Researchers from all statistical backgrounds can use graphical tools to better understand (if not demystify) how the application of various normalization techniques and/or models affect their results. All in all, scientists can gain more confidence in the data analysis pipelines they choose and in the results they draw at the mere cost of briefly creating and exploring graphical outputs during their analyses.

## Conclusions

Modern data analysis is most reliable when models and visuals are used congruently. Unfortunately, there is a propensity for researchers to overtrust model results without confirming them with graphics. This, as we have shown, calls into question the soundness of results derived from differential expression studies. Solving this problem is straightforward and does not require scientists to drastically change their differential expression analyses. Instead, scientists simply need to incorporate effective plotting tools during their usual analysis pipelines. The main motivation of this paper was to provide a collection of examples that show *why* visualization tools should be an integral part of two-group differential expression analysis. We hope our work may motivate researchers to take into account plotting tools that are conveniently and freely available for differential expression analysis. We also hope our work may influence developers to create additional RNA-seq plotting tools that can be applied outside of the case of two-group differential expression. This could include plotting tools for cases that contain many groups, cases of single-cell analysis, and cases where researchers are looking for specificity rather than differential expression.

We strive to serve another small role in this solution with our R software package called “bigPint” that includes the plotting techniques introduced in this paper, many of which are unique additions to the array of plotting tools currently available in differential expression analysis packages. The “bigPint” website is available online [37]. To encourage scientists to use our resource, we include short vignette articles on our website that introduce users to our package. One article provides a recommended pipeline for iterating between models and visualizations when performing differential expression analysis [38]. There is a need to make it easier for researchers to use models and visuals in a complimentary fashion when analyzing RNA-seq data. Our software incorporates data structures that allow users to transition smoothly between our plots and popular models from packages like edgeR [9], DESeq2 [21], and limma [23]. We demonstrate the ease of transition between models and visualizations in the articles of our website. All articles are written using reproducible code that new users can follow. It is our hope that such work will serve a small part in upgrading the RNA-seq analysis world into one that more wholistically extracts meaningful biological information using both models and visuals.

## Methods

Four public RNA-seq datasets were studied in this paper. R [39] was used to conduct analyses. Packages htmlwidgets [40], ggplot2 [41], shiny [42], and plotly [43] were used to build the graphics. The pkgdown [44] package was used to construct the “bigPint” software webpage. Our interactive applications were deployed on shinyapps.io [45].

## Additional files

Additional file 1 Scatterplot matrix for gene cluster that remained as kidney-specific dEGs after tMM normalization. Scatterplot matrix of the 1136 genes that were in the first cluster (of Fig. 17) from genes that remained as kidney-specific DEGs even after TMM normalization. With this scatterplot matrix, we verify from an additional perspective that these genes demonstrate the expected patterns of DEGs.(JPG 140 kb)

Additional file 2 Scatterplot matrix for gene cluster that remained as liver-specific dEGs after tMM normalization. Scatterplot matrix of the 933 genes that were in the first cluster (of Fig. 18) from genes that remained as liver-specific DEGs even after TMM normalization. With this scatterplot matrix, we verify from an additional perspective that these genes demonstrate the expected patterns of DEGs. (JPG 140 kb)

Additional file 3 Scatterplot matrix for gene cluster that were removed from kidney-specific dEGs after tMM normalization. Scatterplot matrix of the 529 genes that were in the first cluster (of Fig. 19) from genes that no longer remained as kidney-specific DEGs after TMM normalization. With this scatterplot matrix, we verify from an additional perspective that these genes do not demonstrate the expected patterns of DEGs too strongly (they do not deviate much from the *x=y* line in the treatment scatterplots). This provides additional evidence that TMM normalization removing these genes from DEG status may be valid. (JPG 135 kb)

Additional file 4 Scatterplot matrix for gene cluster that were added as liver-specific dEGs after tMM normalization. Scatterplot matrix of the 317 genes that were in the first cluster (of Fig. 20) from genes that were added as liver-specific DEGs after TMM normalization. With this scatterplot matrix, we see that the genes do not demonstrate the expected patterns of DEGs too strongly (they do not deviate much from the *x=y* line in the treatment scatterplots). In fact, these pink genes appear similarly to what we saw from the scatterplot matrix of the red genes (Additional file 3). This is somewhat of a surprise, given that the pink genes were *added* by TMM normalization, while the red genes were *removed* by TMM normalization. Stated differently, we would expect the pink genes to appear more like differentially expressed genes if TMM normalization is appropriate, but we could not confirm this expectation. We solved this problem using standardization techniques (Figs. 23 and 24).(JPG 135 kb)

Additional file 5 Example litre plots for genes that remained as kidney-specific dEGs after tMM normalization. Example litre plots from the 1136 genes that were in the first cluster (Fig. 17) of genes that remained as kidney-specific DEGs even after TMM normalization. With these litre plots, we verify from an additional perspective that these genes demonstrate the expected patterns of DEGs.(JPG 666 kb)

Additional file 6 Example litre plots for genes that remained as liver-specific dEGs after tMM normalization. Example litre plots from the 933 genes that were in the first cluster (Fig. 18) from genes that remained as liver-specific DEGs even after TMM normalization. With these litre plots, we verify from an additional perspective that these genes demonstrate the expected patterns of DEGs. (JPG 651 kb)

Additional file 7 Example litre plots for genes that were removed from kidney-specific dEGs after tMM normalization. Example litre plots from the 529 genes that were in the first cluster (Fig. 19) of genes that no longer remained as kidney-specific DEGs after TMM normalization. With these litre plots, we verify from an additional perspective that these genes do not demonstrate the expected patterns of DEGs. This provides additional evidence that TMM normalization removing these genes from DEG status may be valid. (JPG 654 kb)

Additional file 8 Example litre plots for genes that were added as liver-specific dEGs after tMM normalization. Example litre plots from the 317 genes that were in the first cluster (Fig. 20) from genes that were added as liver-specific DEGs after TMM normalization. With these litre plots, we see that the genes do not demonstrate the expected patterns of DEGs in a trustworthy manner. In fact, these pink genes appear similarly to what we saw from the example litre plots of the red genes (Additional file 7). This is somewhat of a surprise, given that the pink genes were *added* by TMM normalization, while the red genes were *removed* by TMM normalization. Stated differently, we would expect the pink genes to appear more like differentially expressed genes if TMM normalization is appropriate, but we could not confirm this expectation. We solved this problem using standardization techniques (Figs. 27 and 28). (JPG 653 kb)

## Data Availability

The bigPint methods used in this paper are available on the software website [37]. The bigPint package is available on Bioconductor at https://bioconductor.org/packages/devel/bioc/html/bigPint.html. The four public datasets discussed in this publication are available online: Three are deposited on the NCBI Sequence Read Archive with accession numbers SRA000299 [12], PRJNA318409 [24], and SRA048710 [22]. One is deposited on the NCBI Gene Expression Omnibus with accession number GSE61857 [27]. Reproducible scripts for all figures in this manuscript are available online at https://github.com/lindsayrutter/VisualizationMethods.

## Abbreviations

DEG: Differentially expressed gene
Litre plot: Replicate treatment plot
MDS: Multidimensional scaling
NGS: Next-Generation sequencing
RNA-seq: RNA-sequencing
TMM: Trimmed mean of M values
YPD: YP-Glucose
