# Supplementary figures and images for: Visualization methods for differential expression analysis
Source: BMC Bioinformatics. 2019 Sep 6;20:458. doi: 10.1186/s12859-019-2968-1 (PMC6731617; doi:10.1186/s12859-019-2968-1)

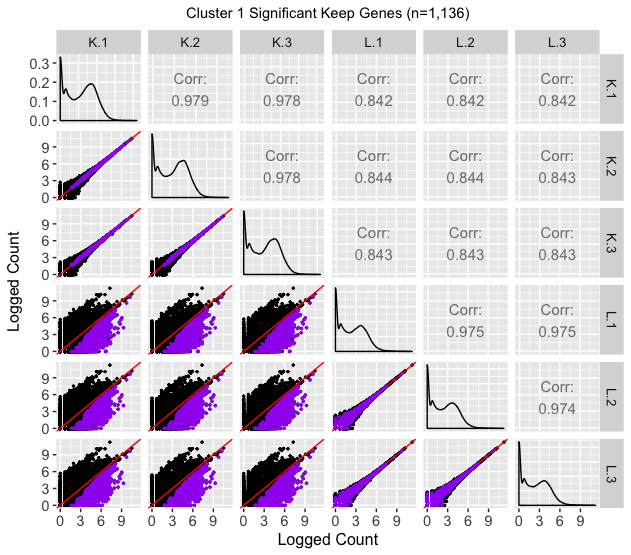

Supplement: Supplementary file 1 — Scatterplot matrix for gene cluster that remained as kidney-specific dEGs after tMM normalization. Scatterplot matrix of the 1136 genes that were in the first cluster (of Fig. 17) from genes that remained as kidney-specific DEGs even after TMM normalization. With this scatterplot matrix, we verify from an additional perspective that these genes demonstrate the expected patterns of DEGs.(JPG 140 kb) [file 12859_2019_2968_MOESM1_ESM.jpg]

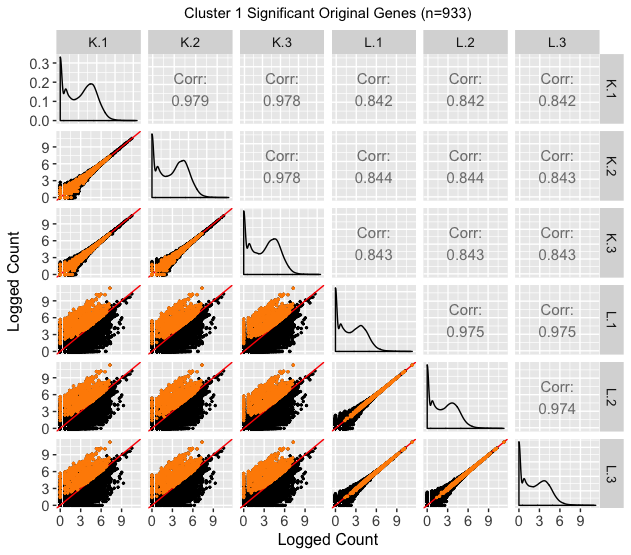

Supplement: Supplementary file 2 — Scatterplot matrix for gene cluster that remained as liver-specific dEGs after tMM normalization. Scatterplot matrix of the 933 genes that were in the first cluster (of Fig. 18) from genes that remained as liver-specific DEGs even after TMM normalization. With this scatterplot matrix, we verify from an additional perspective that these genes demonstrate the expected patterns of DEGs. (JPG 140 kb) [file 12859_2019_2968_MOESM2_ESM.jpg]

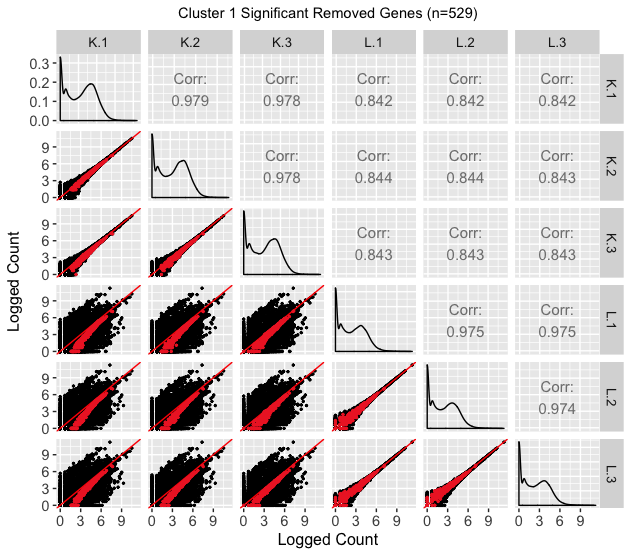

Supplement: Supplementary file 3 — Scatterplot matrix for gene cluster that were removed from kidney-specific dEGs after tMM normalization. Scatterplot matrix of the 529 genes that were in the first cluster (of Fig. 19) from genes that no longer remained as kidney-specific DEGs after TMM normalization. With this scatterplot matrix, we verify from an additional perspective that these genes do not demonstrate the expected patterns of DEGs too strongly (they do not deviate much from the x=y line in the treatment scatterplots). This provides additional evidence that TMM normalization removing these genes from DEG status may be valid. (JPG 135 kb) [file 12859_2019_2968_MOESM3_ESM.jpg]

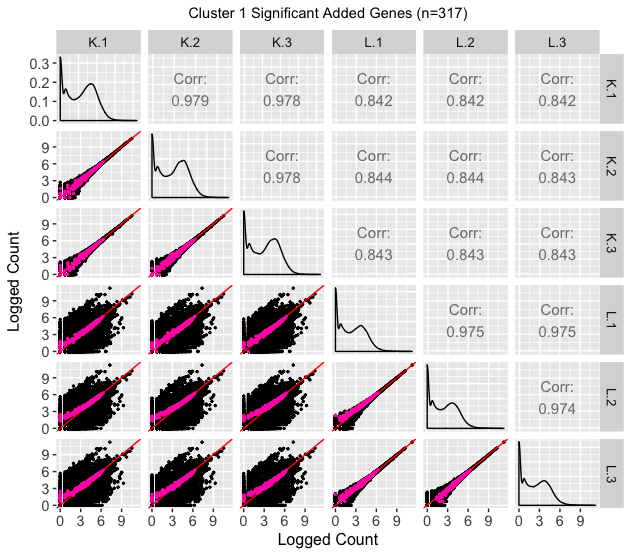

Supplement: Supplementary file 4 — Scatterplot matrix for gene cluster that were added as liver-specific dEGs after tMM normalization. Scatterplot matrix of the 317 genes that were in the first cluster (of Fig. 20) from genes that were added as liver-specific DEGs after TMM normalization. With this scatterplot matrix, we see that the genes do not demonstrate the expected patterns of DEGs too strongly (they do not deviate much from the x=y line in the treatment scatterplots). In fact, these pink genes appear similarly to what we saw from the scatterplot matrix of the red genes (Additional file 3). This is somewhat of a surprise, given that the pink genes were added by TMM normalization, while the red genes were removed by TMM normalization. Stated differently, we would expect the pink genes to appear more like differentially expressed genes if TMM normalization is appropriate, but we could not confirm this expectation. We solved this problem using standardization techniques (Figs. 23 and 24).(JPG 135 kb) [file 12859_2019_2968_MOESM4_ESM.jpg]

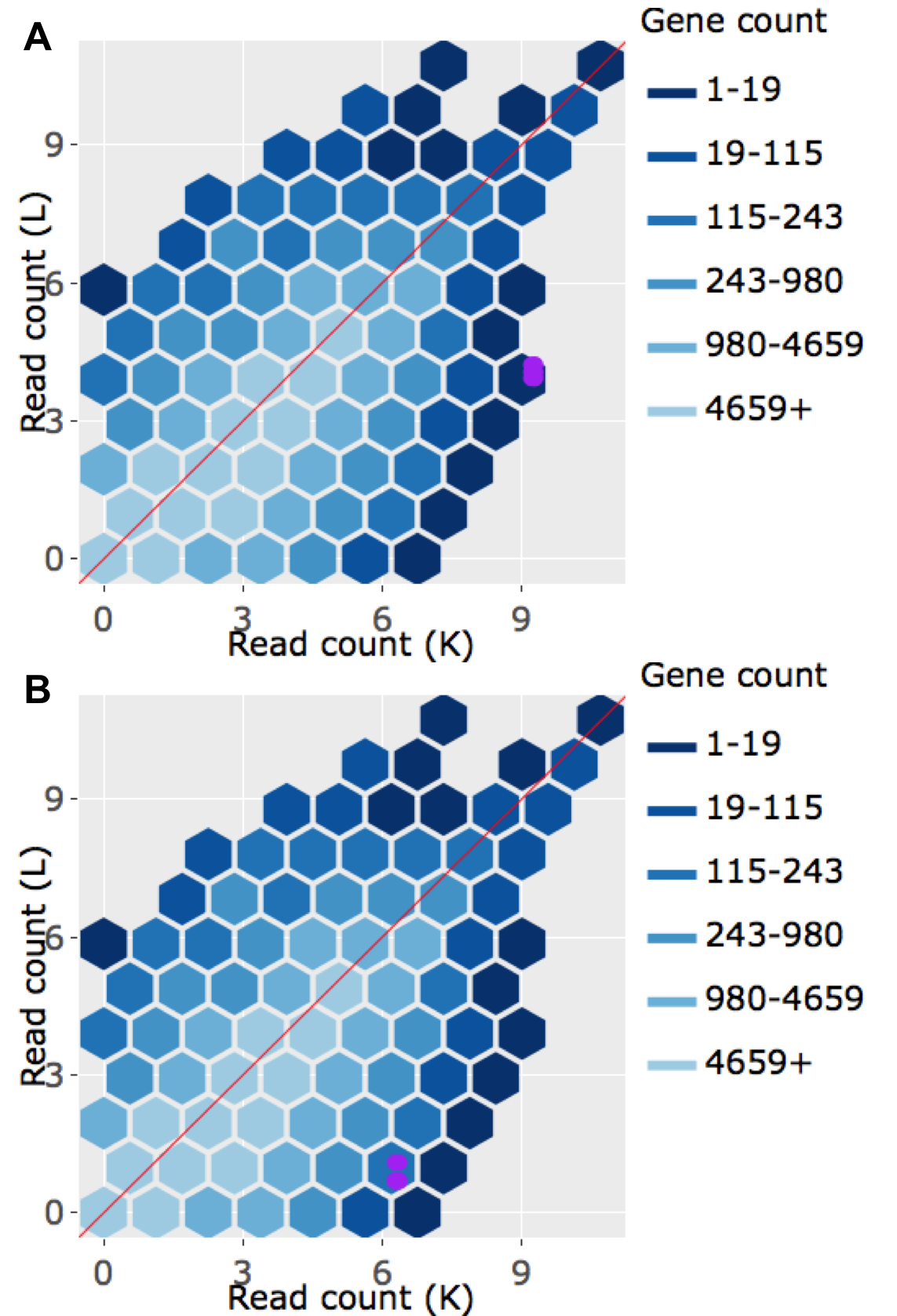

Supplement: Supplementary file 5 — Example litre plots for genes that remained as kidney-specific dEGs after tMM normalization. Example litre plots from the 1136 genes that were in the first cluster (Fig. 17) of genes that remained as kidney-specific DEGs even after TMM normalization. With these litre plots, we verify from an additional perspective that these genes demonstrate the expected patterns of DEGs.(JPG 666 kb) [file 12859_2019_2968_MOESM5_ESM.jpg]

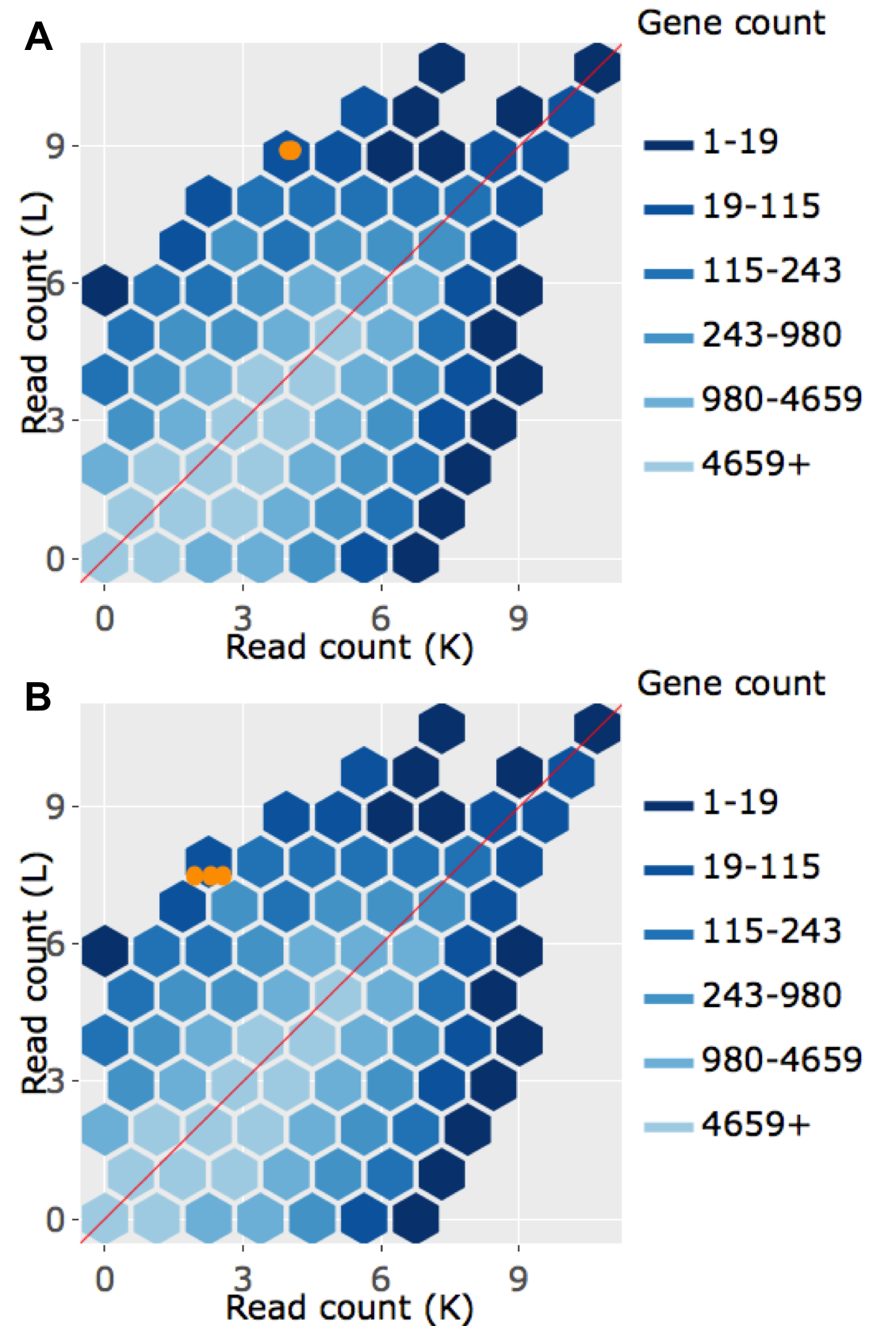

Supplement: Supplementary file 6 — Example litre plots for genes that remained as liver-specific dEGs after tMM normalization. Example litre plots from the 933 genes that were in the first cluster (Fig. 18) from genes that remained as liver-specific DEGs even after TMM normalization. With these litre plots, we verify from an additional perspective that these genes demonstrate the expected patterns of DEGs. (JPG 651 kb) [file 12859_2019_2968_MOESM6_ESM.jpg]

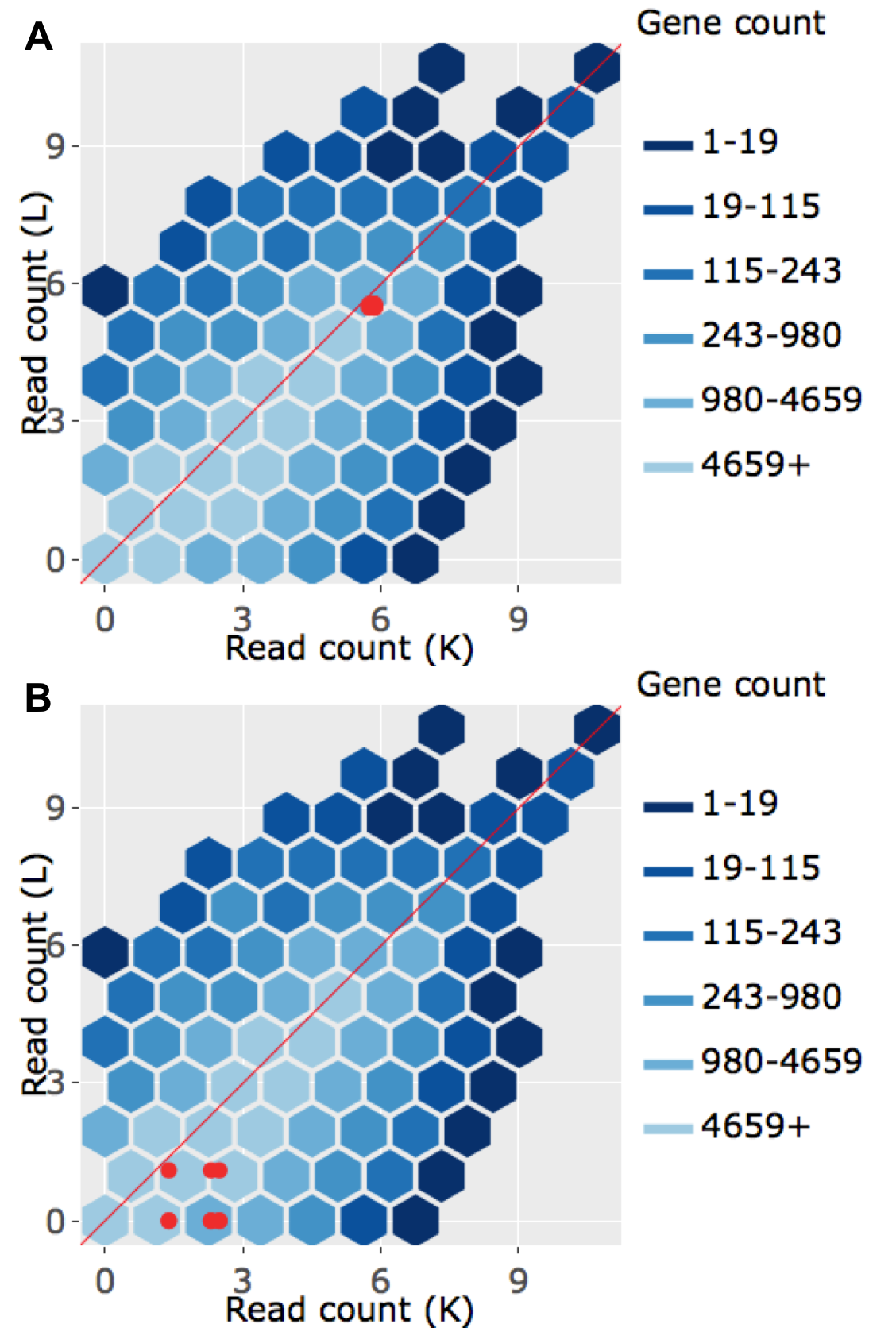

Supplement: Supplementary file 7 — Example litre plots for genes that were removed from kidney-specific dEGs after tMM normalization. Example litre plots from the 529 genes that were in the first cluster (Fig. 19) of genes that no longer remained as kidney-specific DEGs after TMM normalization. With these litre plots, we verify from an additional perspective that these genes do not demonstrate the expected patterns of DEGs. This provides additional evidence that TMM normalization removing these genes from DEG status may be valid. (JPG 654 kb) [file 12859_2019_2968_MOESM7_ESM.jpg]

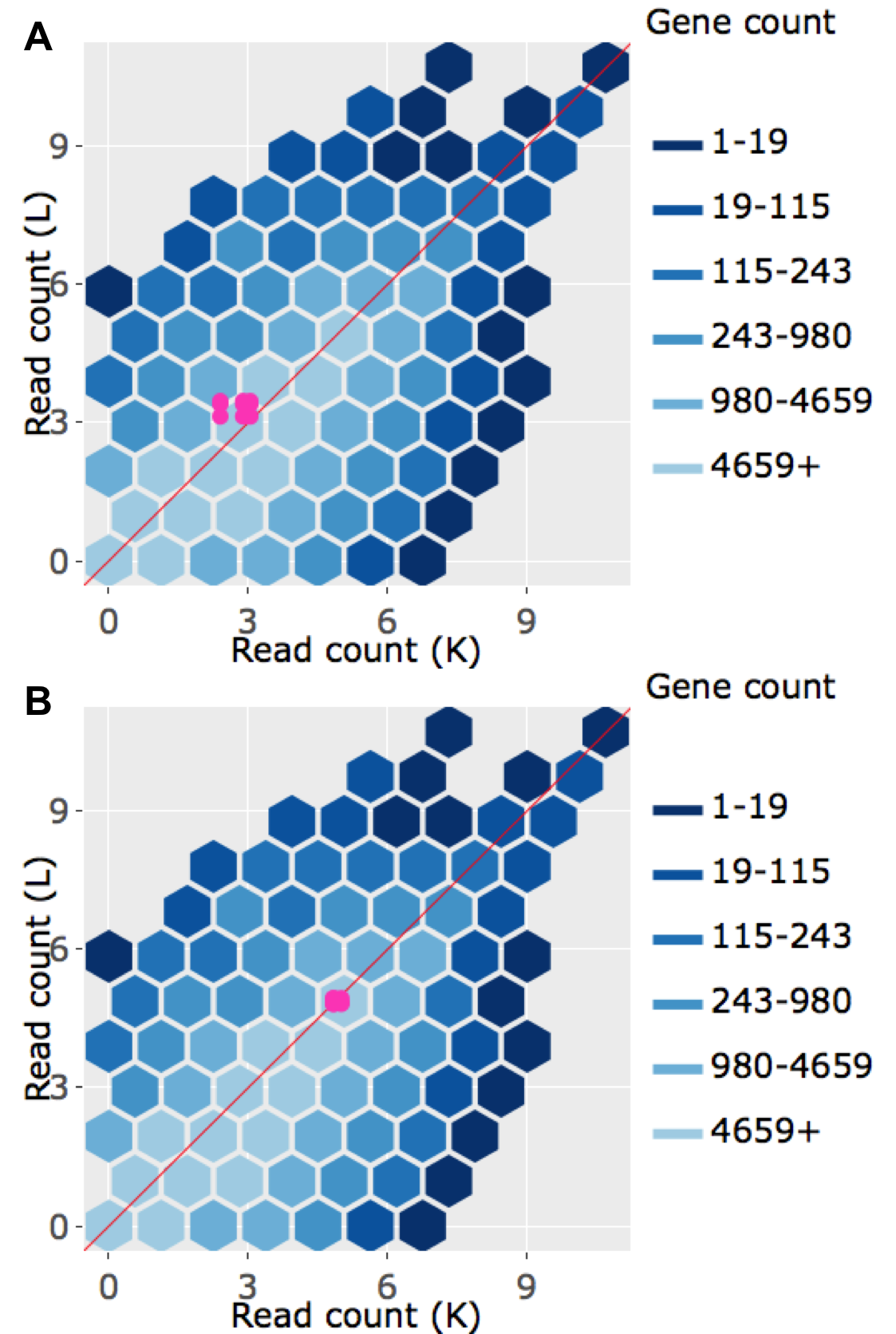

Supplement: Supplementary file 8 — Example litre plots for genes that were added as liver-specific dEGs after tMM normalization. Example litre plots from the 317 genes that were in the first cluster (Fig. 20) from genes that were added as liver-specific DEGs after TMM normalization. With these litre plots, we see that the genes do not demonstrate the expected patterns of DEGs in a trustworthy manner. In fact, these pink genes appear similarly to what we saw from the example litre plots of the red genes (Additional file 7). This is somewhat of a surprise, given that the pink genes were added by TMM normalization, while the red genes were removed by TMM normalization. Stated differently, we would expect the pink genes to appear more like differentially expressed genes if TMM normalization is appropriate, but we could not confirm this expectation. We solved this problem using standardization techniques (Figs. 27 and 28). (JPG 653 kb) [file 12859_2019_2968_MOESM8_ESM.jpg]
